# The Role of the [2Fe‐2S] Cluster of *Escherichia coli* IscR in Responding to Redox‐Cycling Agents

**DOI:** 10.1111/mmi.70021

**Published:** 2025-09-11

**Authors:** Rajdeep Banerjee, Erin L. Mettert, Angela S. Fleischhacker, Patricia J. Kiley

**Affiliations:** ^1^ Department of Biomolecular Chemistry University of Wisconsin‐Madison Madison Wisconsin USA

**Keywords:** Fe‐S cluster biogenesis, Fe‐S cluster homeostasis, redox regulation, Rrf2 family of transcription factors

## Abstract

The mechanisms by which cells respond to growth inhibitory redox‐cycling agents is only partially understood. In *Escherichia coli* K12, the IscR regulon, which includes the ISC and SUF Fe‐S cluster biogenesis machineries, is differentially expressed in response to these agents. Here, we report how one redox‐cycling agent, phenazine methosulfate (PMS), regulates IscR activity via its [2Fe‐2S] cluster cofactor. A direct role for IscR in mediating the response to PMS was inferred from the PMS‐dependent weakening of [2Fe‐2S]‐IscR binding to an *isc* operon type 1 DNA site in vitro. This decrease in DNA binding was attributed to the accompanying oxidation of its [2Fe‐2S]^1+^ cluster. Exposure of anaerobic cultures to PMS leads to increased *isc* expression, as expected from IscR cluster oxidation and impaired binding to type 1 sites in the *isc* promoter. However, this same anaerobic PMS treatment did not change expression of type 2 site promoters, such as *suf*, which require IscR that lacks an Fe‐S cluster (apo‐IscR) for effective transcriptional regulation. In contrast, PMS exposure under aerobic conditions significantly increased both *isc* and *suf* expression, indicating the formation of both [2Fe‐2S]^2+^‐IscR and apo‐IscR. This effect was partially attributed to superoxide generation by PMS under aerobic conditions, as evidenced by a superoxide dismutase‐deficient mutant showing a modest impact on *isc* and *suf* expression. Together, these findings provide new insights into redox‐cycling dependent regulation of IscR activity and highlight the distinct activities of apo‐IscR, [2Fe‐2S]^2+^‐IscR and [2Fe‐2S]^1+^‐IscR in controlling the IscR regulon.

## Graphical abstract

Redox cycling agents like phenazine methosulfate (PMS) are known for upsetting cellular redox balance. Here we describe how PMS leads to upregulation of the ISC and SUF Fe‐S cluster biogenesis pathways in *Escherichia coli* when PMS oxidizes the [2Fe‐2S]^1+^ cluster of the transcription factor IscR, thereby derepressing the operon encoding ISC and, when a sufficient pool of apo‐IscR accumulates, activating expression of SUF.

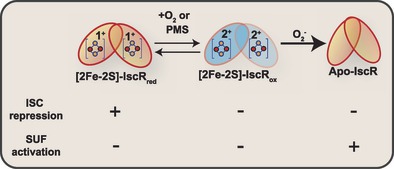

## 1 Introduction

IscR is a global transcription factor that plays a central role in maintaining cellular Fe‐S cluster homeostasis in *Escherichia coli* and other bacteria by regulating the expression of two major Fe‐S cluster biogenesis pathways, ISC (Iron Sulfur Cluster) and SUF (Sulfur Utilization Factor), along with other cellular functions (Mettert and Kiley 2014, 2024). Under conditions of increased Fe‐S cluster demand, IscR shifts from repressing the *isc* operon to activating the *suf* operon, resulting in the upregulation of both pathways (Nesbit et al. 2009; Giel et al. 2013, 2006; Schwartz et al. 2001). While this adaptation is essential for meeting cellular Fe‐S cofactor requirements for Fe‐S cluster cofactors under a variety of conditions, the precise molecular mechanisms by which IscR coordinates this response remain incompletely understood.

Biochemical and structural studies of wild‐type and variant IscR proteins have provided a mechanistic framework for understanding IscR function (Fleischhacker et al. 2012; Giel et al. 2013; Nesbit et al. 2009; Rajagopalan et al. 2013). Anaerobically isolated wild‐type IscR forms a dimer, with each monomer coordinating a [2Fe‐2S] cluster (Fleischhacker et al. 2012). When bound to this cluster, IscR represses the *isc* operon, which encodes both IscR and the ISC biogenesis machinery (Schwartz et al. 2001; Giel et al. 2013; Fleischhacker et al. 2012). In contrast, apo‐IscR that is devoid of any cluster—either due to ligand mutation or chemical chelation—is inactive for regulating *isc* transcription. However, this apo‐form *is* functional for activating expression of the *suf* operon, encoding the alternate SUF Fe‐S biogenesis pathway (Lee et al. 2004, 2008; Yeo et al. 2006; Nesbit et al. 2009; Outten et al. 2004; Giel et al. 2013). The preference for regulation by the apo‐form versus the [2Fe‐2S] form of IscR is determined by the type of DNA binding motif found in the promoter region (Giel et al. 2013, 2006; Chowdhury et al. 2021). The *isc* promoter features type 1 motifs that specifically bind [2Fe‐2S]‐IscR, whereas the *suf* promoter contains a type 2 site that binds either [2Fe‐2S]‐IscR or apo‐IscR. Notably, despite binding both forms of IscR, type 2 motif‐containing promoters require apo‐IscR for productive transcriptional regulation through an unknown mechanism (Nesbit et al. 2009, 2012; Chowdhury et al. 2021; Rajagopalan et al. 2013).

Expression of both *isc* and *suf* operons is higher in cells grown under aerobic conditions compared to anaerobic growth, suggesting that O_2_ availability regulates IscR activity (Giel et al. 2006). Mössbauer and EPR spectroscopic analyses of IscR have shown that in the absence of O_2_, IscR predominantly contains a reduced [2Fe‐2S]^1+^ cluster, whether in whole cells or after protein isolation (Fleischhacker et al. 2012; Schwartz et al. 2001). Upon exposure to O_2_, the cluster is oxidized to the [2Fe‐2S]^2+^ state. However, cluster oxidation did not appear to affect [2Fe‐2S]‐IscR binding affinity to the *iscR* promoter (Schwartz et al. 2001; Fleischhacker et al. 2012), nor did O_2_ lead to significant decomposition of the cluster making the mechanism of regulation different from the well‐known Fe‐S cluster O_2_ sensor, FNR (Mettert and Kiley 2018; Crack et al. 2012). Therefore, a new model emerged proposing that increased *isc* expression under aerobic conditions results from reduced synthesis of [2Fe‐2S]‐IscR rather than cluster oxidation and degradation (Giel et al. 2013). Under conditions of increased Fe‐S cluster demand, such as aerobiosis, IscR is hypothesized to compete less efficiently with other client proteins for access to the Fe‐S biogenesis machinery. As a result, reduced levels of [2Fe‐2S]‐IscR lead to partial derepression of *isc* expression and the resultant increase in apo‐IscR activates *suf* expression, forming a coordinated response to meeting Fe‐S demands (Mettert and Kiley 2014; Giel et al. 2013). In addition, [2Fe‐2S]‐IscR relies primarily on the ISC Fe‐S biogenesis pathway for cluster assembly, suggesting a direct link between IscR cluster occupancy and a negative feedback loop regulating *isc* operon expression (Schwartz et al. 2001; Giel et al. 2013; Mettert and Kiley 2014).

IscR has also been implicated in the upregulation of the *isc* and *suf* operons in response to redox‐cycling agents. While these small molecules can be synthetically derived, they are produced naturally by a variety of bacteria, plants, fungi, and archaea (Dietrich and Kiley 2011; Jacob et al. 2011). Phenazines are a well‐known example of redox‐cycling agents synthesized and excreted by different *Pseudomonas* and *Streptomyces* species (Pierson and Pierson 2010). *E. coli* and other enterobacteria do not produce redox‐cycling agents but may be routinely exposed to these compounds in natural, polymicrobial environments (Sheplock et al. 2013). These compounds share a common feature in that they are readily reduced and re‐oxidized under physiological conditions. This redirecting of electrons to new targets can disrupt normal electron flow and thereby has the potential to impair cellular processes (Dietrich and Kiley 2011; Jacob et al. 2011). Furthermore, redox‐cycling agents elevate intracellular superoxide levels (Hassan and Fridovich 1979), which can destabilize Fe‐S clusters in dehydratase enzymes (Flint, Emptage, et al. 1993; Flint, Tuminello, and Emptage 1993; Imlay 2006), thereby increasing the demand for Fe‐S biogenesis. Transcriptomic studies revealed that the addition of the redox‐cycling agent paraquat elevated RNA levels of both the *isc* and *suf* operons, although the responsible transcription factor was not initially identified (Blanchard et al. 2007). Other experiments using *lacZ* fusions or RNA measurements of *isc* or *suf o*peron expression suggested that IscR is required for the cellular response to a different redox‐cycling agent, phenazine methosulfate (PMS) (Lee et al. 2008; Gerstel et al. 2020; Yeo et al. 2006). However, it remains unclear whether these agents act indirectly—by increasing Fe‐S demand—or directly—by inactivating [2Fe‐2S]‐IscR either through oxidative modification similar to the redox sensor SoxR (Dietrich and Kiley 2011; Gu and Imlay 2011; Kobayashi et al. 2025; Lee et al. 2015; Sheplock et al. 2013; Singh et al. 2013) or through cluster destabilization by superoxide.

In this work we investigated the role of IscR in the response of *E. coli* to the redox‐cycling agent PMS. To test whether the previously reported upregulation of the *isc* and *suf* operons by redox‐cycling agents (Blanchard et al. 2007; Gerstel et al. 2020; Yeo et al. 2006) is linked to changes in IscR Fe‐S cluster occupancy, we measured the stability of the [2Fe‐2S] cluster and its impact on DNA binding in the presence of PMS or a superoxide generating system using in vitro assays. We also analyzed IscR‐dependent gene expression in *E. coli* treated with PMS under both aerobic and anaerobic conditions, correlating these findings with in vitro DNA binding results. Contrary to previous findings (Fleischhacker et al. 2012), we observed that O_2_ alone was sufficient to inactivate [2Fe‐2S]‐IscR binding to a type 1 DNA motif. Furthermore, PMS was as effective as O_2_ in decreasing DNA binding of [2Fe‐2S]‐IscR, suggesting a direct effect of PMS on IscR function, similar to that of SoxR (Gu and Imlay 2011). However, treatment with PMS under aerobic growth conditions resulted in increased expression of apo‐IscR dependent promoters, suggesting a more complex regulatory mechanism involving redox‐cycling agents during aerobic growth.

## 2 Results

### 2.1 IscR Plays a Key Role in Mediating the Response to the Redox‐Cycling Agent, PMS

**FIGURE 1:**
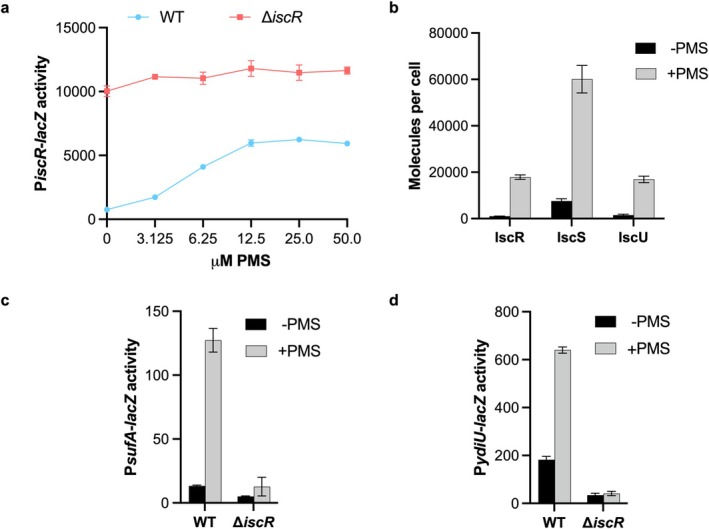
PMS induces changes in IscR‐mediated gene expression. (a) β‐galactosidase activity from P*iscR*‐*lacZ* (expressed in Miller units) in wild‐type (WT; blue) and ∆*iscR* (red) strains was measured after aerobic cultures were grown in LB to an OD_600_ of 0.1 and were either left untreated or exposed to indicated PMS concentrations for 1 h. (b) Quantitative Western blot analysis of IscR, IscS, and IscU levels in a WT strain grown aerobically in LB to an OD_600_ of 0.1 and either left untreated (black) or exposed to 12.5 μM PMS for 1 h (gray). The average protein molecules per cell ± standard error from three experimental replicates in untreated and PMS‐treated cultures were respectively 1035 ± 93 and 17864 ± 950 for IscR; 7556 ± 989 and 60117 ± 5917 for IscS; and 1525 ± 382 and 16895 ± 1387 for IscU. (c and d) β‐galactosidase activity from P*sufA*‐*lacZ* and P*ydiU*‐*lacZ*, respectively, in strains grown as described in panel b.

Previous studies have shown that redox‐cycling agents induce expression of the *isc* and *suf* operons (Blanchard et al. 2007; Yeo et al. 2006; Gerstel et al. 2020). To characterize the role of IscR in this response in vivo, we assessed the effect of the redox‐cycling agent PMS on *isc* expression using a transcriptional reporter (P*iscR*‐*lacZ*) as *iscR* is the first gene in the *isc* operon and by quantifying IscR, IscS, and IscU protein levels. Treating aerobically grown cells for 1 h with increasing PMS concentrations revealed that 12.5 μM PMS resulted in maximal derepression of P*iscR*‐*lacZ*, increasing expression 8‐fold (Figure 1a). No induction by PMS was observed in a strain lacking IscR, confirming its essential role in the response. The level of derepression in PMS‐treated IscR^+^ cells was approximately half the amount observed in the *iscR* deletion strain, suggesting a small fraction of IscR remains active under these conditions. Quantitative Western blot analysis showed a 17‐fold increase in IscR levels in cells exposed to 12.5 μM PMS compared to untreated controls (Figure 1b and Figure S1a), consistent with P*iscR* derepression. Similarly, protein levels of other operon members, IscS and IscU, also increased significantly with 12.5 μM PMS (Figure 1b, Figure S1b,c), further supporting enhanced expression of the *isc* operon.

We hypothesized that during this 1 h period, PMS decreases the pool of active [2Fe‐2S]‐IscR, leading to *isc* operon derepression. The corresponding increase in IscR levels, presumed to be mostly apo‐IscR, led us to test whether expression of apo‐IscR activated promoters containing a type 2 motif was also increased. Indeed, expression of the *sufA* and *ydiU* promoters was both elevated upon PMS addition under aerobic conditions, and this increase in expression was dependent on IscR (Figure 1c,d). For P*sufA*, the P*sufA*‐*lacZ* reporter assayed lacked the OxyR binding site (Outten et al. 2004; Zheng et al. 2001), ruling out OxyR as the cause of PMS‐induced expression. Similarly, Fur‐mediated derepression was not responsible for P*sufA* induction by PMS since a P*sufA*‐*lacZ* variant lacking an intact Fur binding site (Outten et al. 2004) showed a comparable increase in expression upon PMS treatment (Figure S2a) and Fur‐mediated repression of a synthetic Fur reporter (P*fepB*syn‐*lacZ*) (Beauchene et al. 2017) was not significantly affected by PMS (Figure S2b). Collectively, these findings suggest that redox‐cycling agents modulate IscR activity.

**FIGURE 2:**
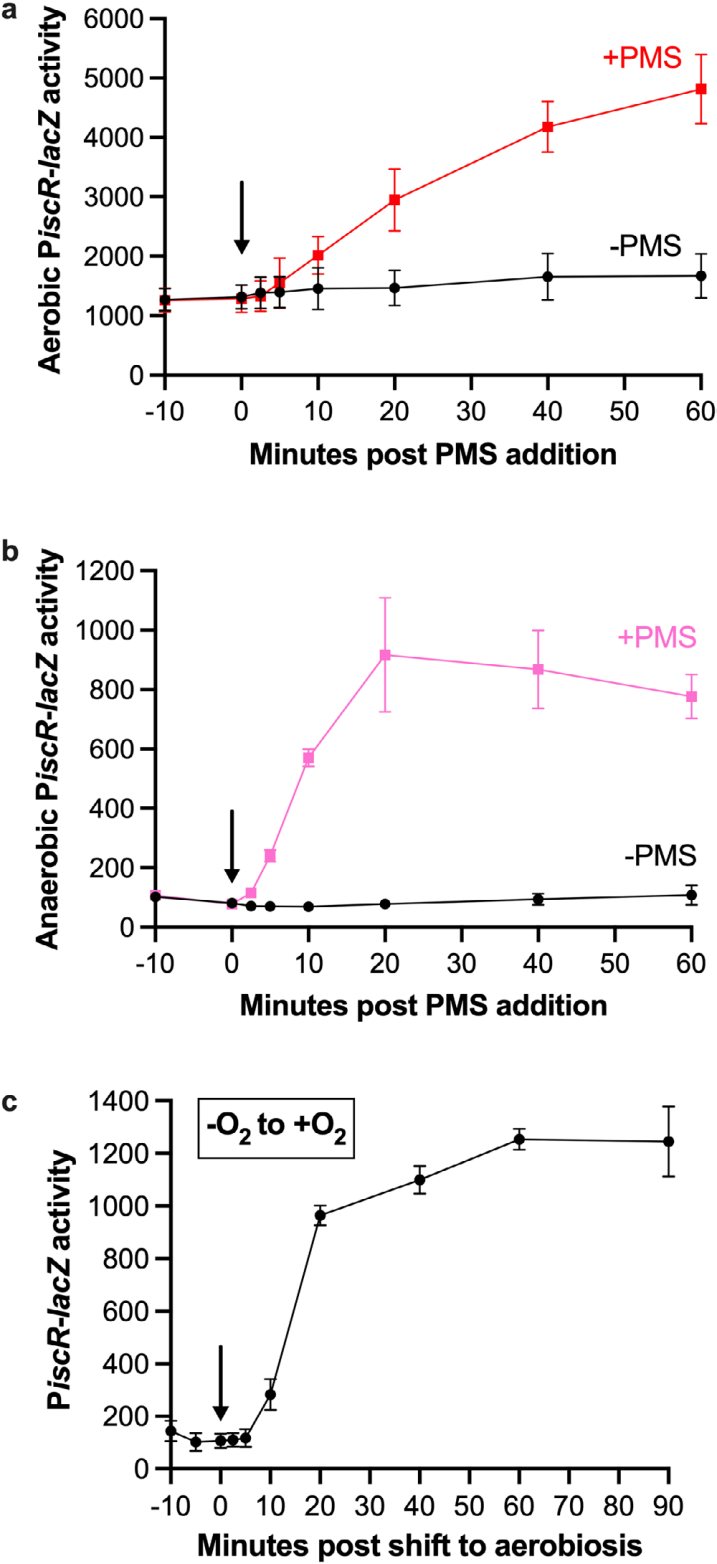
IscR responds rapidly upon exposure of cells to PMS or O_2_. β‐galactosidase activity from P*iscR*‐*lacZ* (expressed in Miller units) was measured over time in (a) aerobic and (b) anaerobic cultures grown in LB to an OD_600_ of 0.1 and either left untreated or exposed to 12.5 μM PMS, and in (c) anaerobic cultures that were grown in LB to an OD_600_ of 0.1 and then exposed to O_2_. The arrow in each panel indicates the time (0 min) in which the culture was exposed to either PMS or O_2_. For each experiment, β‐galactosidase activity in a Δ*iscR* mutant was also measured, averaging 5246 ± 750 and 9138 ± 1096 Miller units before and after 60 min PMS treatment under aerobic conditions; 2702 ± 76 and 4003 ± 186 before and after 60 min PMS treatment under anaerobic conditions; and 2998 ± 53 and 6353 ± 244 before and after O_2_ exposure, indicating that O_2_ and PMS had minor effects on P*iscR*‐*lacZ* expression in the absence of IscR.

To determine the speed at which IscR responds to PMS in vivo, we performed a time‐course experiment. Aerobically grown cells were treated with 12.5 μM PMS, and P*iscR*‐*lacZ* activity was measured at various intervals over a 1 h period. P*iscR* expression increased within 5 to 10 min of PMS addition (Figure 2a). Given this rapid effect of PMS on IscR‐dependent gene expression, we hypothesized that PMS or superoxide produced by PMS in vivo (Hassan and Fridovich 1979) acts on a pre‐existing pool of [2Fe‐2S]‐IscR to decrease its activity.

### 2.2 Superoxide, but Not PMS, Alters Stability of [2Fe‐2S] Cluster of IscR

**FIGURE 3:**
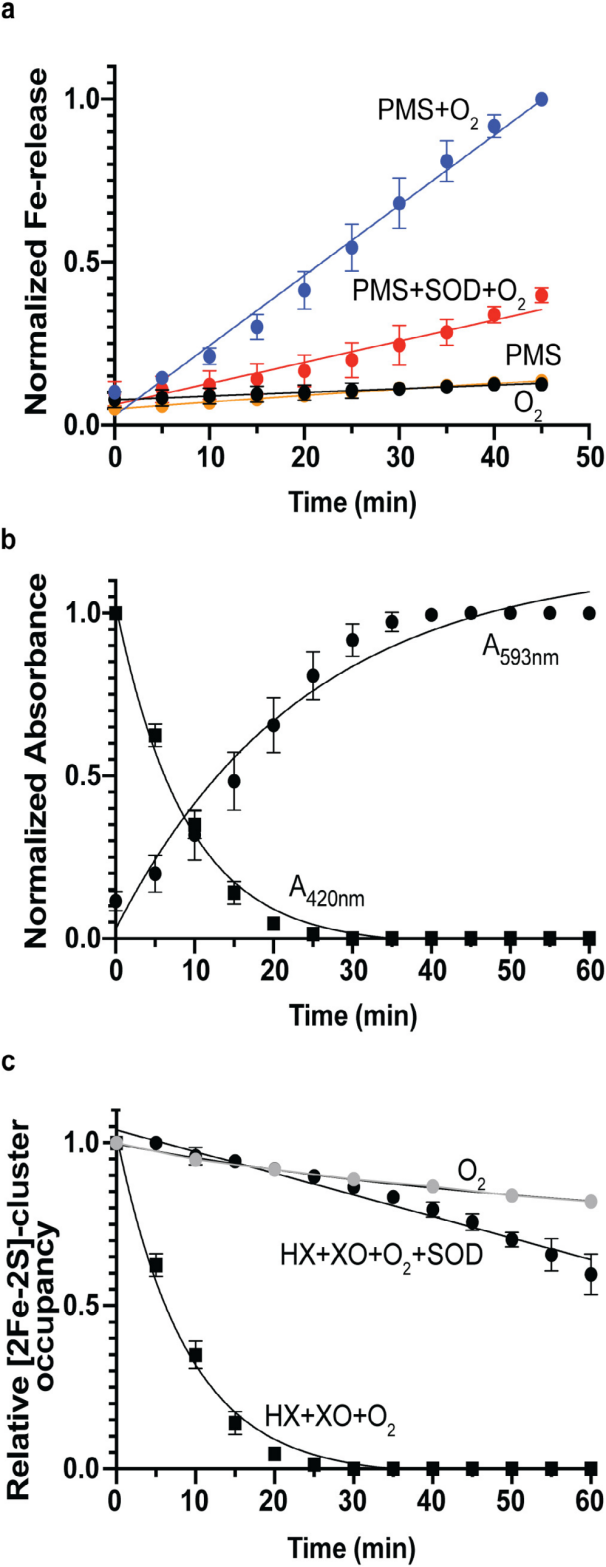
IscR [2Fe‐2S] cluster stability to oxidants in vitro. Cluster stability of anaerobically isolated [2Fe‐2S]‐IscR (5 μM in 10 mM potassium phosphate [pH 7.4], 200 mM KCl) was measured at 25°C as indicated. (a) Released iron was determined by the A_593nm_ in the presence of 1 mM ferene under the following conditions: air (orange); 20 μM PMS (black); 20 μM PMS with air (blue); and 20 μM PMS, air, and 30 U Cu,Zn SOD (red). The A_593nm_ was normalized to the value for ~100% of cluster iron released, represented as 1 on the *y*‐axis. (b) [2Fe‐2S] cluster loss with air, 100 μM hypoxanthine and 5 mU of xanthine oxidase was measured by iron release with ferene as described in (a) (circles) and as the normalized A_420nm_ replotted from (c) (squares). (c) Relative [2Fe‐2S]‐cluster occupancy was measured under the following conditions: Air (gray circles); air, 100 μM hypoxanthine and 5 mU of xanthine oxidase (black squares); and air, 100 μM hypoxanthine, 5 mU of xanthine oxidase and 30 U of Cu,Zn SOD (black circles). Relative [2Fe‐2S]‐cluster occupancy was calculated as the ratio of A_420nm_ from each spectrum (representative spectra are shown in Figure S4a,b) at each time point relative to its value at time *t* = 0 min.

To test whether the PMS‐dependent decrease in [2Fe‐2S]‐IscR activity in vivo could be explained by destabilization of the [2Fe‐2S] cluster, we measured cluster loss in vitro by the absorbance of iron bound‐ferene at 593 nm. Following treatment of anaerobically isolated [2Fe‐2S]^1+^‐IscR (5 μM) with 20 μM PMS, no change in absorption at 593 nm (A_593_) was observed, indicative of intact Fe‐S clusters (Figure 3a). In contrast, introducing air in addition to PMS resulted in an increase in absorption at 593 nm, indicating iron release and cluster destabilization. We reason that this destabilization resulted from superoxide formed from the reaction of PMS with O_2_, as iron release was significantly inhibited when the superoxide scavenger, superoxide dismutase (SOD), was added to the assay and air alone did not increase the absorption at 593 nm (Figure 3a). Because PMS requires a source of electrons to produce superoxide in this reaction, we propose that the reduced [2Fe‐2S] cluster of IscR acts as an electron donor, although other possibilities cannot be excluded. Taken together, these findings suggest that superoxide, but not PMS alone, promotes cluster loss from IscR.

To provide additional evidence for the role of superoxide in cluster degradation, we used the well‐established superoxide generating system of air, hypoxanthine, and xanthine oxidase (Flint, Tuminello, and Emptage 1993). When anaerobically isolated [2Fe‐2S]^1+^‐IscR was exposed to the superoxide generating system, a time‐dependent increase in the A_593_ was observed, indicating iron release (Figure 3b). Maximal iron release, corresponding to nearly 100% of the cluster iron, occurred within 30 min. Iron release was dependent on the superoxide generating system, as air alone resulted in no increase in A_593_ (Figure 3a). Cluster loss was also assayed by monitoring changes in the characteristic UV–visible spectrum of the [2Fe‐2S] cluster of IscR (Figure 3b,c, Figure S4a). As was shown in previous EPR and Mössbauer studies (Fleischhacker et al. 2012; Schwartz et al. 2001), exposing anaerobically isolated [2Fe‐2S]‐IscR to air for ~5 min results in cluster oxidation from [2Fe‐2S]^1+^ to [2Fe‐2S]^2+^. Over longer time periods, the 420 nm absorbance of the oxidized [2Fe‐2S] cluster showed only minor decreases, indicating slow cluster degradation in air (Figure 3c) (Figure S4b). In contrast, exposure of [2Fe‐2S]‐IscR to the superoxide generating system resulted in spectral changes indicative of accelerated cluster loss, mirroring the A_593_ associated with iron release in the ferene assay (Figure 3b,c). The rate of cluster degradation due to superoxide, which was calculated to be 4.8 × 10^5^ M^−1^ s^−1^, was more rapid than IscR exposed to air (Figure S3, Table S1). Addition of SOD slowed the rate to levels comparable with air alone, confirming that superoxide specifically accelerated cluster degradation (Figure 3c).

Since some H_2_O_2_ is also likely produced in these in vitro systems, we examined whether H_2_O_2_ can also lead to [2Fe‐2S]‐cluster degradation by directly adding H_2_O_2_ to IscR aerobically. We found that H_2_O_2_ degrades the cluster more slowly than superoxide, as only ~30% of the cluster was lost after treatment with 100 μM H_2_O_2_ for 20 min (Figure S4c,e,f, Table S1). Addition of the H_2_O_2_ scavenger, catalase, protected the cluster from degradation (Figure S4d,e). Thus, these in vitro data indicate that superoxide and, to a lesser extent, H_2_O_2_ promote cluster degradation as compared to air.

### 2.3 DNA Binding by [2Fe‐2S]‐IscR Is Inhibited by PMS

**FIGURE 4:**
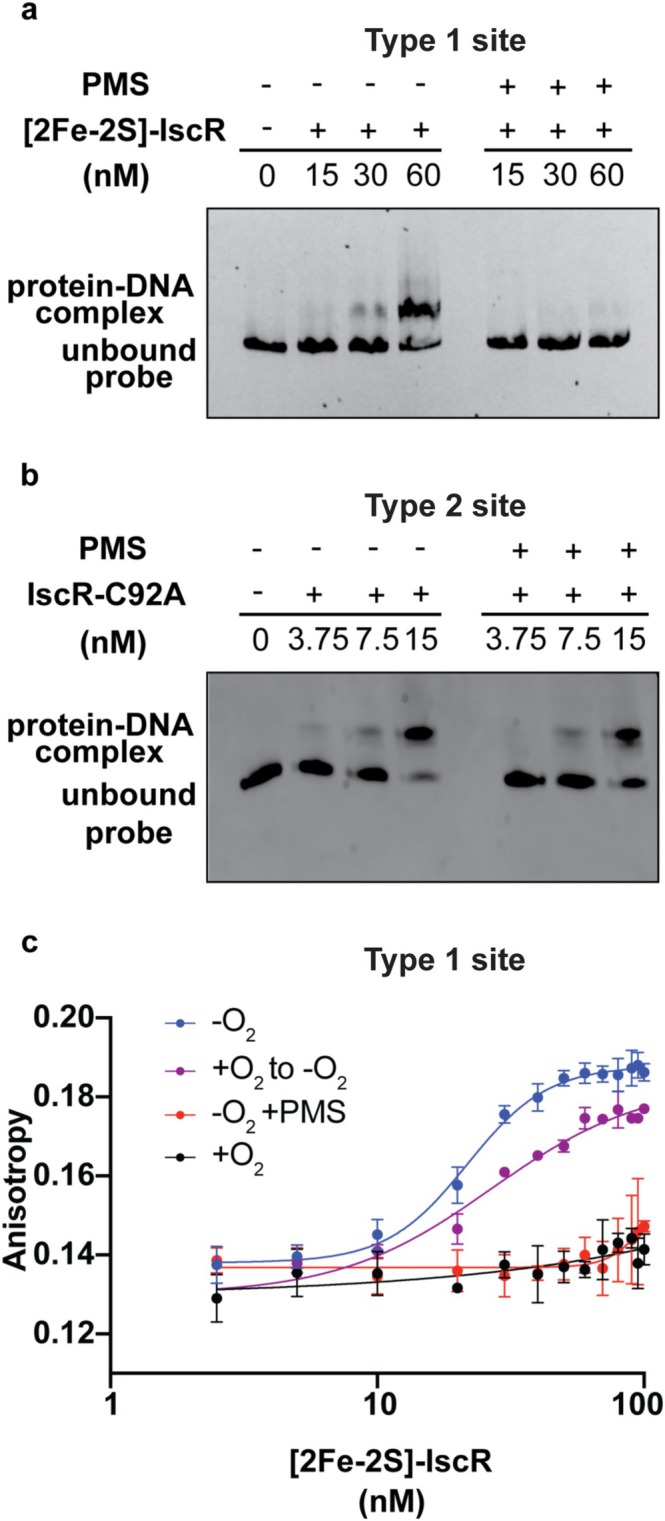
PMS affects DNA binding by [2Fe‐2S]‐IscR. (a) EMSA of anaerobically isolated [2Fe‐2S]‐IscR incubated with a type 1 IscR binding site (4.5 nM), either in the presence or absence of 240 nM PMS under anaerobic conditions. (b) EMSA of IscR(C92A) incubated with a type 2 IscR binding site (6 nM), either in the presence or absence of 240 nM PMS under anaerobic conditions. (c) Fluorescence anisotropy‐derived DNA binding isotherms to a type 1 site by [2Fe‐2S]‐IscR assayed anaerobically after 15 min treatment under the following conditions: Anaerobic (blue); anaerobic and 2 μM PMS (red); and air (purple). [2Fe‐2S]‐IscR was also exposed to air for 15 min and then assayed in air (black).

Since our in vitro data suggested that PMS does not lead to degradation of [2Fe‐2S]‐cluster of IscR under anaerobic conditions, we hypothesized that PMS should not affect its DNA binding activity under these conditions. However, to our surprise, electrophoretic mobility shift assays (EMSAs) under anaerobic conditions showed that PMS treatment of anaerobically isolated [2Fe‐2S]^1+^‐IscR impaired binding to a type 1 site compared to the untreated control (Figure 4a). To determine whether PMS was specifically acting on the [2Fe‐2S] form of IscR, we performed the EMSA under the same conditions using IscR(C92A), a variant lacking any cluster, which is functional for binding a type 2 IscR binding motif (Nesbit et al. 2009). We found that PMS did not affect the DNA binding activity of IscR(C92A) compared to the untreated control (Figure 4b), indicating that PMS does not nonspecifically impede DNA binding. To quantify the effect of PMS on [2Fe‐2S]‐IscR DNA binding affinity, we measured IscR binding to fluorescently labeled DNA containing a type 1 site using fluorescence anisotropy under anaerobic conditions. Addition of PMS weakened the apparent DNA binding affinity of anaerobically isolated [2Fe‐2S]^1+^‐IscR by at least 50‐fold (untreated and PMS‐treated IscR had an apparent *K*_*d*_ of 22 nM and > 1000 nM, respectively; Figure 4c). Since the ferene results showed that PMS did not cause cluster degradation, but rather possible cluster oxidation (Figure 3a), we considered the possibility that PMS can oxidize the [2Fe‐2S]^1+^ cluster to the [2Fe‐2S]^2+^ state leading to impaired DNA binding by IscR. An impact of cluster oxidation on DNA binding was unexpected because our previous studies with air‐oxidized IscR indicated that oxidation of [2Fe‐2S]^1+^‐IscR to the [2Fe‐2S]^2+^ cluster did not alter IscR DNA binding affinity (Fleischhacker et al. 2012). To understand this possible discrepancy, we measured DNA binding of IscR exposed to air for 15 min, a time period sufficient to oxidize the [2Fe‐2S]^1+^ cluster to [2Fe‐2S]^2+^ cluster (Fleischhacker et al. 2012) and assayed DNA binding under aerobic conditions. We found a similar impairment in IscR DNA binding as anaerobic PMS‐treated IscR (Figure 4c). However, when DNA binding of air‐oxidized protein was measured under anaerobic conditions in our Coy chamber as previously reported (Fleischhacker et al. 2012), the binding affinity (*K*_*d*_ = 26 nM) was nearly restored to that of untreated protein (Figure 4c), suggesting, quite unexpectedly, that the [2Fe‐2S]^2+^ cluster of IscR was likely auto‐reduced to [2Fe‐2S]^1+^ state. Taken together, our findings highlight the importance of cluster oxidation state of IscR in regulating DNA binding of type 1 sites under in vitro conditions.

### 2.4 [2Fe‐2S]‐IscR Responds Similarly to O_2_ or PMS Under Anaerobic Growth Conditions

The above results predict that the addition of O_2_ or PMS to anaerobically grown cells should oxidize the [2Fe‐2S]^1+^ cluster of IscR and lead to derepression of the type 1 site regulated *iscR* promoter. To test this hypothesis, we performed a time course experiment and measured P*iscR*‐*lacZ* activity after adding 12.5 μM PMS to anaerobic cultures. IscR responded rapidly to PMS since P*iscR*‐*lacZ* expression increased within 5 to 10 min of PMS addition (Figure 2b), similar to the response time observed when aerobic cells were exposed to PMS (Figure 2a). These data indicate that PMS alone is sufficient to oxidize and inactivate [2Fe‐2S]^1+^‐IscR in vivo. Similarly, shifting anaerobically grown cells to aerobic growth conditions also resulted in an increase in transcription from P*iscR* within 5 to 10 min of introducing O_2_ (Figure 2c), suggesting that in cells, oxidation of the IscR [2Fe‐2S]^1+^ cluster by PMS or O_2_ occurs at a similar, rapid rate.

Importantly, a new steady state of *isc* expression, indicative of re‐establishing a small pool of [2Fe‐2S]^1+^‐IscR, was observed at ~20 min post PMS treatment under anaerobic conditions (Figure 2b) and after anaerobic cells were exposed to O_2_ (Figure 2c). However, for PMS treatment under aerobic growth conditions, *isc* expression had not yet reached a new steady state by 60 min, and expression was 6‐fold higher than that of anaerobically treated cells at 60 min (Figure 2a).

### 2.5 An In Vivo Role for Superoxide

**FIGURE 5:**
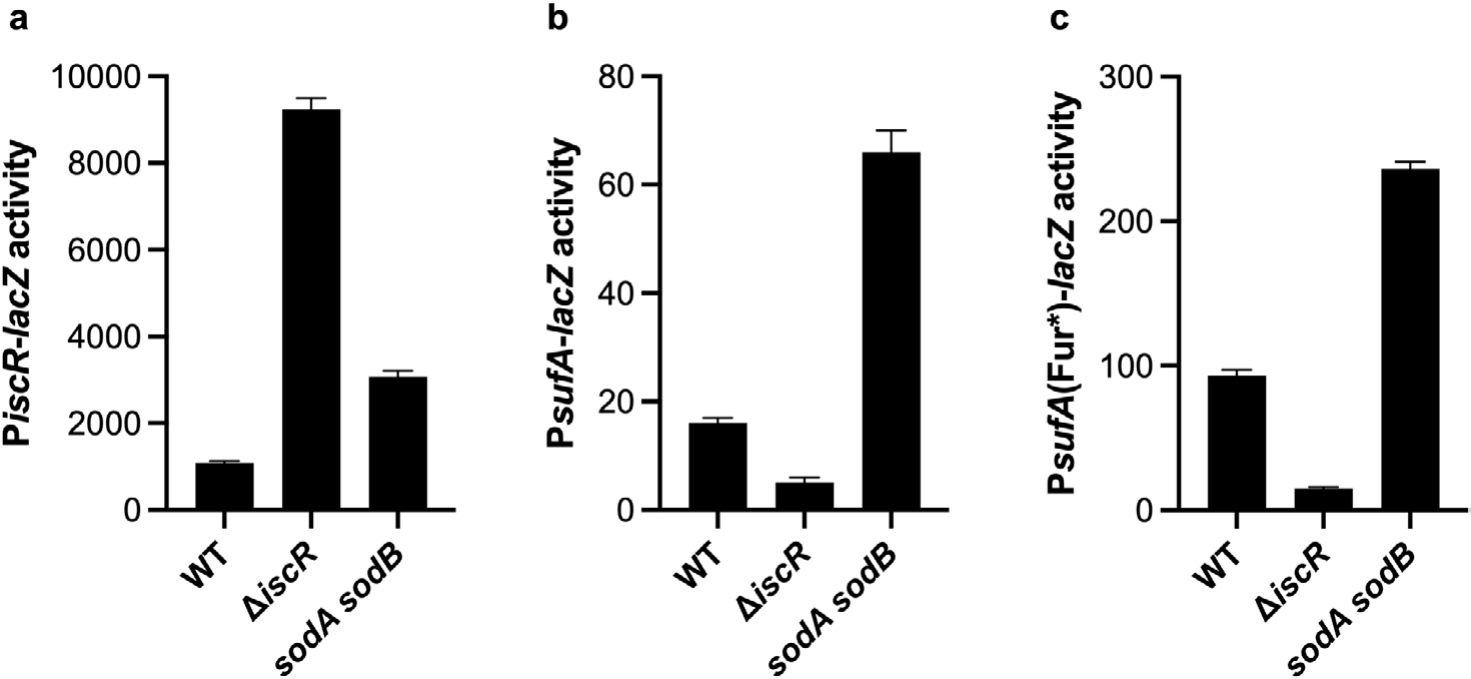
[2Fe‐2S]^1+^‐IscR and apo‐IscR activities are respectively decreased and increased in a mutant defective in eliminating superoxide. β‐galactosidase activity (expressed in Miller units) from (a) P*iscR*‐*lacZ*, (b) P*sufA*‐*lacZ*, and (c) P*sufA*(Fur*)‐*lacZ* (in which the Fur binding site was mutated) was measured in WT, Δ*iscR*, and *sodAsodB* strains grown aerobically in LB.

Since PMS is known to elevate superoxide levels in aerobic cells (Hassan and Fridovich 1979), we considered the possibility that under aerobic growth conditions, the oxidized [2Fe‐2S]^2+^ cluster was destabilized by superoxide, as suggested by our in vitro data (Figure 3), contributing to the additive effect of aerobic conditions and PMS in Figure 2a and decreasing the pool of [2Fe‐2S]^1+^‐IscR. To test whether superoxide levels alone had an effect on IscR activity, we assayed a *sodAsodB* mutant (*sodA25*::Mu*dPR13* Δ*sodB*), which has elevated superoxide levels because it lacks cytoplasmic superoxide dismutase enzymes, SodA and SodB (Djaman et al. 2004; Gu and Imlay 2011). P*iscR*‐*lacZ* expression increased ~3‐fold in this mutant compared to that in the wild‐type strain under standard aerobic growth (Figure 5a). A similar increase in expression was observed in the *sodAsodB* mutant for apo‐IscR activated P*sufA*‐*lacZ* or P*sufA*(Fur*)*‐lacZ*, its variant lacking an intact Fur binding site (Figure 5b,c). Together, these findings suggest that superoxide makes a measurable contribution to increasing the pool of apo‐IscR under aerobic conditions. While the effect of superoxide could be direct by promoting cluster degradation, it is also possible that competition between IscR and other Fe‐S protein substrates for the Fe‐S cluster assembly machinery, which likely intensifies in the presence of superoxide (Imlay 2006), may also contribute to delayed [2Fe‐2S]^1+^‐IscR synthesis, an accumulation of apo‐IscR, and an altered steady state of *isc* transcription under aerobic growth conditions with PMS.

### 2.6 Candidates for a [2Fe‐2S]^2+^‐IscR Reducing System

Collectively, our data indicate that cluster oxidation state plays an important role in regulating [2Fe‐2S]‐IscR activity, which is similar to the regulatory mechanism established for [2Fe‐2S]‐SoxR. For SoxR, it has been demonstrated that *rsxABCDGE*, *rseC*, and *apbE* gene products form a complex that reduces the oxidized [2Fe‐2S]^2+^ cluster using the reductant NAD(P)H (Lee et al. 2024, 2022; Koo et al. 2003). To address whether this reducing system also plays a role in reducing the IscR [2Fe‐2S]^2+^ cluster, P*iscR*‐*lacZ* expression was measured in a strain lacking *rsxC* and *rseC*. We observed a minor (2‐fold) increase in expression in this mutant compared to wild‐type under both anaerobic and aerobic conditions (Figure S5a), suggesting that RsxC and RseC do not significantly contribute to IscR [2Fe‐2S] cluster reduction. Furthermore, IscR activity was not affected in a mutant lacking *fpr*, encoding a flavodoxin/ferredoxin‐NADP^+^ reductase (Figure S5a). Finally, although a mutant lacking the ferredoxin encoded by the *iscRSUAhscBAfdx* operon exhibits a significant defect in [2Fe‐2S]^1+^‐IscR activity under both anaerobic (Figure S5b) and aerobic conditions (Figure S5b and Giel et al. 2013), it is difficult to distinguish whether this is due to defective IscR [2Fe‐2S] cluster reduction or a general disruption in cellular Fe‐S cluster biogenesis.

### 2.7 Cluster Oxidation Appears to Primarily Affect IscR‐Dependent Regulation of Type 1 Motifs

Our results show that oxidation of the IscR [2Fe‐2S]^1+^ cluster inactivates IscR binding to its type 1 sites within P*iscR*. We next tested whether the oxidized cluster form([2Fe‐2S]^2+^‐IscR) is capable of regulating promoters with type 2 motifs. This was addressed by comparing the anaerobic activities of [2Fe‐2S]^1+^‐IscR, [2Fe‐2S]^2+^‐IscR, and apo‐IscR in transcriptional regulation of type 2 promoter reporters, the IscR‐repressed P*hyaA*syn‐*lacZ* and the IscR‐activated P*sufA*(Fur*)‐*lacZ*, as compared to the type 1 promoter, P*iscR*‐*lacZ*. Since IscR levels are subject to negative autoregulation (Giel et al. 2013), strains were constructed to produce equivalent IscR levels under anaerobic conditions by placing *iscR* under the control of P*tac* and inducing with 160 μM isopropyl‐β‐d‐thiogalactopyranoside (IPTG) (Giel et al. 2013). The well‐characterized clusterless variant, *iscR(C92A)*, was used as a proxy for apo‐IscR (Nesbit et al. 2012; Giel et al. 2013).

**FIGURE 6:**
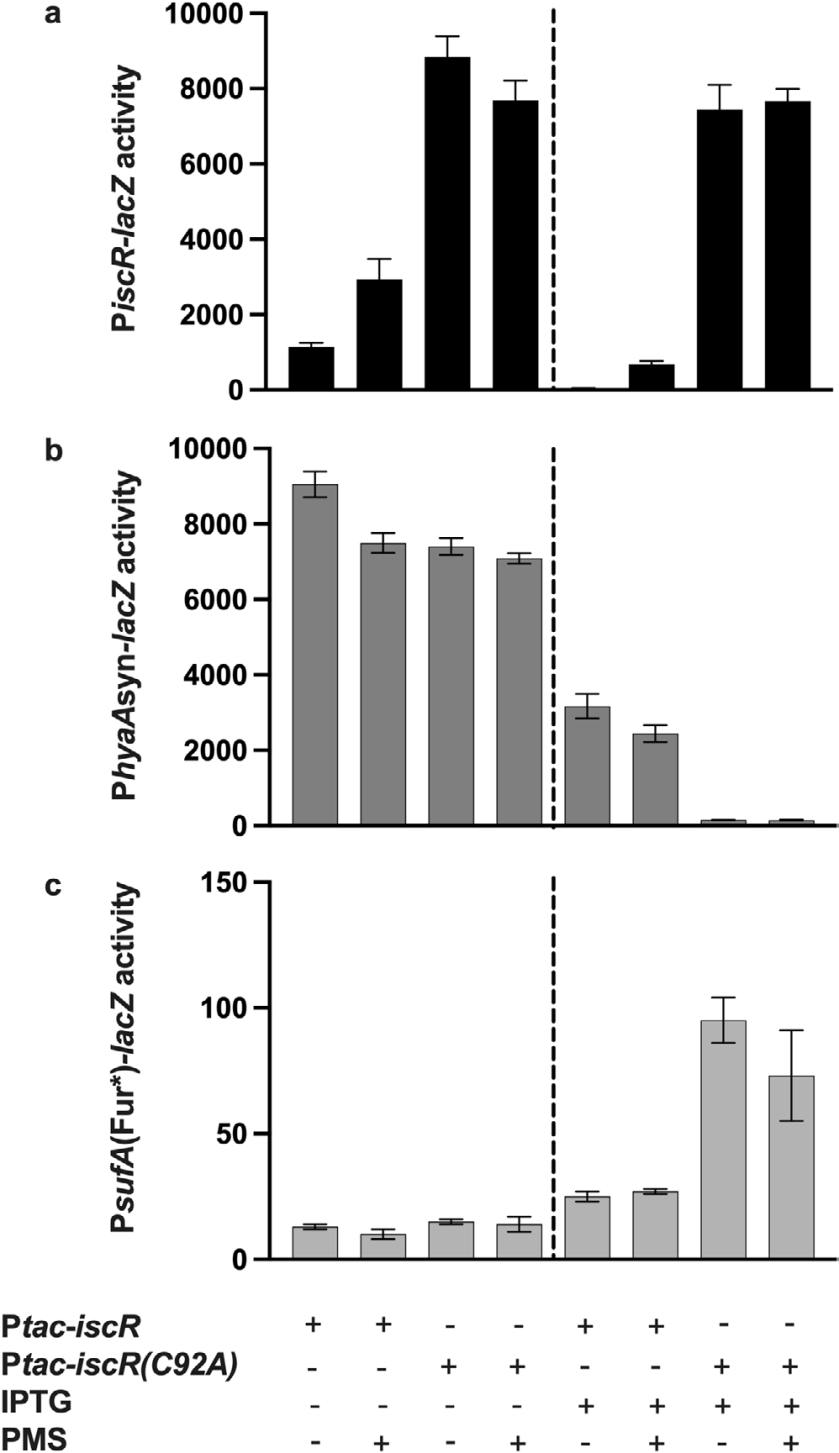
Oxidation of the IscR [2Fe‐2S]^1+^ cluster primarily affects regulation of type 1 motifs. To compare transcriptional regulation by [2Fe‐2S]^1+^‐IscR, [2Fe‐2S]^2+^‐IscR, and apo‐IscR, β‐galactosidase activity was assayed in strains containing (a) the type 1 promoter reporter P*iscR*‐*lacZ*, and the type 2 promoter reporters (b) P*hyaA*syn‐*lacZ* and (c) P*sufA*(Fur*)‐*lacZ* (in which the Fur site was mutated). Strains also contained either *iscR* or the apoprotein variant *iscR(C92A)* under control of P*tac* and were grown anaerobically in MOPS minimal medium containing 20 mM arabinose, 0.2% casamino acids, and 10 μg/mL tetracycline to an OD_600_ of 0.1. Where indicated, cultures included 160 μM IPTG and/or 12.5 μM PMS.

As expected, expression of P*iscR*‐*lacZ* was maximally repressed under anaerobic conditions when cultures contained IPTG, representing the pool of [2Fe‐2S]^1+^‐IscR produced at this IPTG concentration (Figure 6a). In contrast, PMS‐treated cells showed an intermediate amount of repression of P*iscR*‐*lacZ* (4‐fold), compared to full repression in the untreated cells (32‐fold) and the absence of repression in the control strain containing apo‐IscR [*iscR(C92A)*], indicating a PMS‐dependent partial shift to the oxidized pool of [2Fe‐2S]^2+^‐IscR. As expected, the type 2 regulated promoters, P*hyaA*syn‐*lacZ* and P*sufA*(Fur*)‐*lacZ*, showed a strong preference for apo‐IscR in enhancing their repression and activation, respectively, compared to WT IscR (Figure 6b,c) (Nesbit et al. 2012, 2009; Lee et al. 2008). Furthermore, shifting the pool of [2Fe‐2S]^1+^‐IscR to the oxidized state by PMS addition did not alter expression of the type 2 promoters, P*hyaA* and P*sufA* (Figure 6b,c), unlike the type 1 promoter (Figure 6a). Therefore, these data suggest that oxidation of the [2Fe‐2S]^1+^ cluster to [2Fe‐2S]^2+^ state primarily affects IscR regulation of promoters with type 1 motifs.

### 2.8 Contributions of IscR to Mitigating Oxidative Stress Caused by PMS

**FIGURE 7:**
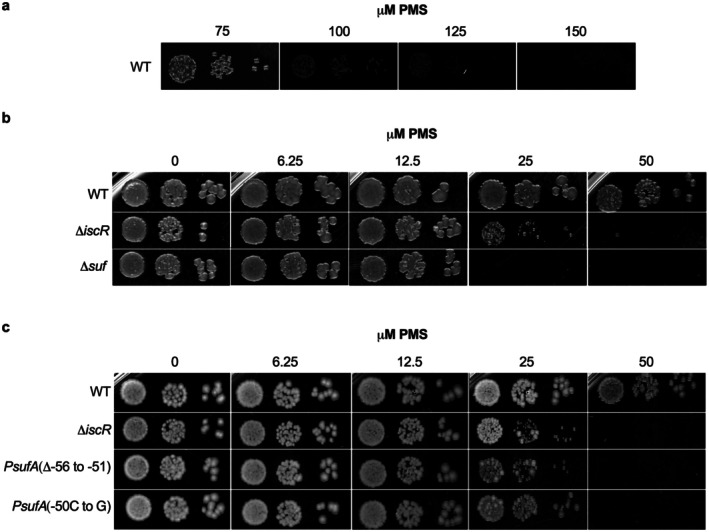
IscR mitigates oxidative stress through transcriptional activation of *suf*. Serial dilutions of wild‐type (WT) or mutant strains grown in LB to an OD_600_ of 0.2 were plated on Tryptone‐Yeast Extract (TYE) agar containing indicated concentrations of PMS. (a) Plates containing high concentrations of PMS (150 μM) were used to test the limit of viability for a WT strain. (b) The viability of strains lacking *iscR* or the *suf* operon was compared to that of WT. (c) The viability of strains containing mutations in the IscR type 2 binding site within P*sufA* (either [Δ−56 to −51] or [−50C to G] relative to the +1 transcription start site) were compared to that of WT and Δ*iscR*.

To compare the impact of PMS‐mediated induction of the IscR regulon on aerobic growth, colony‐forming units were compared for various strain backgrounds plated on 2‐fold increments of increasing concentrations of PMS (Figure 7). The wild‐type parent strain loses viability at 150 μM PMS (Figure 7a), whereas the strain lacking IscR was more sensitive, losing viability at 50 μM PMS (Figure 7b,c). Previous studies have shown that *suf* contributes significantly to PMS resistance (Gerstel et al. 2020), and indeed, we observed that deletion of the *suf* operon resulted in a loss of viability at 25 μM PMS (Figure 7b). This raised the question as to whether PMS sensitivity of the Δ*iscR* mutant was due to the lack of *suf* activation by IscR. Analysis of P*sufA* mutants that are defective in IscR‐binding (Mettert and Kiley 2014) displayed similar PMS sensitivity as the Δ*iscR* mutant (Figure 7c), suggesting that a reduction in *suf* expression is a major contributor to the PMS sensitivity of the Δ*iscR* strain. Furthermore, these findings indicate an important role of IscR in mitigating oxidative stress caused by PMS. Interestingly, the Δ*iscR* and Δ*suf* mutants also displayed PMS sensitivity when plates were incubated under anaerobic conditions (Figure S6), albeit at 2‐fold higher concentrations of PMS compared to those under aerobic conditions (Figure 7b). This observation implies that IscR and Suf are vital to combating PMS‐induced stress even under conditions in which superoxide would not be present.

## 3 Discussion

Fe‐S clusters are crucial protein cofactors that facilitate a wide variety of reactions across the biological world (Beinert 2000; Fontecave 2006). Although Fe‐S clusters are relatively stable under anaerobic conditions, certain cluster types become destabilized under aerobic growth conditions by O_2_, hydrogen peroxide, or superoxide, leading to a deficit of some Fe‐S proteins (Imlay 2008, 2006, 2013; Flint, Emptage, et al. 1993; Flint, Tuminello, and Emptage 1993; Bruska et al. 2013, 2015). This study investigated the mechanism by which the superoxide‐producing redox‐cycling agent PMS leads to upregulation of the *isc* and *suf* operons under aerobic conditions, thus increasing the supply of Fe‐S cluster biogenesis machinery for rebuilding Fe‐S proteins in response to such oxidative stress conditions. We discovered that PMS alone directly inactivates the central homeostatic regulator of Fe‐S cluster biogenesis, IscR, by oxidizing its [2Fe‐2S]^1+^ cluster. Under aerobic conditions, cluster oxidation by PMS triggers a series of events leading to the upregulation of both the SUF and ISC Fe‐S cluster biogenesis pathways. In contrast, under anaerobic conditions, the effect of PMS on IscR is mostly restricted to upregulation of only the ISC Fe‐S biogenesis pathway. Altogether, these findings revealed new insight into how IscR activity is regulated in vivo and its impact in maintaining Fe‐S cluster homeostasis in response to redox‐cycling agents.

### 3.1 Oxidation of the [2Fe‐2S]^1+^ Cluster of IscR Leads to Loss of DNA Binding to Type 1 Sites

Our in vitro analyses and anaerobic growth assays showed that reduced [2Fe‐2S]^1+^‐IscR is the most effective in binding to type 1 sites in the *isc* promoter and repressing its transcription. In contrast, oxidation of [2Fe‐2S]^1+^ cluster to the [2Fe‐2S]^2+^ cluster—either by O_2_ or PMS—was sufficient to weaken DNA binding affinity to a type 1 site in vitro and the repression of the *isc* promoter in vivo. This result was surprising because a previous study (Fleischhacker et al. 2012) concluded that O_2_‐induced oxidation of the cluster did not alter DNA binding affinity. However, as shown here, the expectation that the cluster would remain in the oxidized state when IscR was assayed under anaerobic conditions without added reducing agent was apparently incorrect. Fe‐S cluster “auto‐reduction” has been previously reported for a small number of proteins but is not well understood (Karagas et al. 2014). Nevertheless, the drop in DNA binding affinity by [2Fe‐2S]^2+^‐IscR explains how the *isc* promoter is rapidly derepressed in response to O_2_ or PMS alone, elevating expression of the *isc* operon.

### 3.2 The [2Fe‐2S]^1+^ Cluster of IscR Plays a Key Role in Responding to Redox‐Cycling Agents

The finding that the [2Fe‐2S]^1+^ cluster of IscR can be directly oxidized by a redox‐cycling agent is similar to findings with the redox sensor, SoxR (Dietrich and Kiley 2011; Gu and Imlay 2011; Kobayashi et al. 2025; Singh et al. 2013). Oxidation of the [2Fe‐2S]^1+^ clusters of dimeric SoxR by redox‐cycling agents leads to transcription activation of *soxS* (Ding and Demple 1997; Hidalgo et al. 1997); SoxS in turn induces expression of a regulon that contributes to protection against oxidative damage (Dietrich and Kiley 2011). Both oxidized and reduced SoxR bind a DNA sequence overlapping the promoter elements, but only the oxidized [2Fe‐2S]^2+^ SoxR apparently introduces a DNA distortion that realigns the promoter elements to promote transcription activation of *soxS*, following the paradigm of MerR family members. How oxidation of the [2Fe‐2S]^1+^ cluster of SoxR transmits a conformational change to remodel binding to promoter DNA is currently unknown (Kobayashi 2017; Kobayashi et al. 2011, 2025).

While cluster oxidation of *E. coli* SoxR was initially attributed to cellular superoxide produced by redox‐cycling agents, the leading role of superoxide was questioned when Gu et al. found that there was very little induction of *soxS* transcription in a strain that has elevated endogenous superoxide due to the absence of cytosolic superoxide dismutases (Gu and Imlay 2011). Furthermore, activation of SoxR by redox‐cycling agents was also observed under anaerobic conditions, which was further enhanced by adding a terminal electron acceptor to amplify redox‐cycling, leading the authors to conclude that SoxR is directly regulated by redox‐cycling agents through the oxidation of the [2Fe‐2S]^1+^ cluster. Presumably, the in vivo inactivation we observed of IscR under anaerobic conditions by PMS occurs by the same cluster oxidation mechanism and is similarly limited by the amount of redox‐cycling.

Furthermore, the direct effect of redox‐cycling agents proposed for SoxR is further buttressed by studies of SoxR orthologs from other bacteria and direct in vitro studies (Lee et al. 2015; Kobayashi et al. 2025; Sheplock et al. 2013; Singh et al. 2013). Although the [2Fe‐2S] cluster of SoxR also undergoes reversible oxidation–reduction, in vivo the cluster appears to be maintained in its reduced state through the action of the RsxABCDGE and RseC reducing system (Lee et al. 2024, 2022; Koo et al. 2003) and thus genes under the control of SoxRS are not known to be O_2_ regulated, in contrast to what has been observed with IscR (this study and Giel et al. 2006). While we found only a small effect of deleting the Rsx/RseC system on IscR activity, it is unclear whether such a system is even required to maintain IscR in its reduced state under anaerobic conditions. However, neither O_2_ nor PMS/O_2_ leads to complete inactivation of IscR under the growth conditions tested here, suggesting that in vivo there may be a reducing system capable of maintaining a small pool of reduced [2Fe‐2S]^1+^ cluster of IscR when O_2_ is present. Thus, determining whether there is a dedicated system to reduce the oxidized [2Fe‐2S] cluster of IscR and how this system might contribute to the regulatory circuit of IscR control remains an outstanding question.

DNA binding of another Rrf2 family member, [2Fe‐2S]‐RsrR from *Streptomyces venezuelae*, is also regulated by oxidation–reduction (Crack et al. 2020; Volbeda et al. 2019). In contrast to IscR, oxidation of the [2Fe‐2S]^1+^ cluster of RsrR promotes DNA binding and transcriptional regulation. When the cluster is reduced, a histidine residue becomes protonated and a tryptophan side chain is reoriented, causing dissociation from the DNA. Similar to our observation with IscR, the authors found that O_2_ alone can oxidize the [2Fe‐2S]^1+^ cluster of RsrR in vitro, although less is known about what signals regulate RsrR activity in vivo. Thus, reversible oxidation–reduction of [2Fe‐2S] clusters appears to be an emerging mechanism to regulate this class of transcription factors. As in *E. coli*, many other diverse bacteria exploit IscR to regulate expression of Fe‐S cluster biogenesis pathways in response to oxidative stress (Mettert and Kiley 2024), suggesting the mechanism of cluster oxidation for IscR is likely conserved. Indeed, during review of this manuscript, demonstration that oxidation of *Yersinia entericola* [2Fe‐2S]‐IscR resulted in a significant decrease in binding to the *iscR* promoter was reported (Gray et al. 2025). Furthermore, in *Pseudomonas aeruginosa* , not only did *isc* expression increase upon exposure to the redox‐cycling drugs plumbagin, paraquat, and menadione (Romsang et al. 2014), but also upon exposure to pyocyanin, which is a redox‐cycling compound naturally produced by this bacterium (Meirelles and Newman 2018).

### 3.3 PMS Treatment Leads to Accumulation of Apo‐IscR Under Aerobic but Not Anaerobic Growth Conditions

**FIGURE 8:**
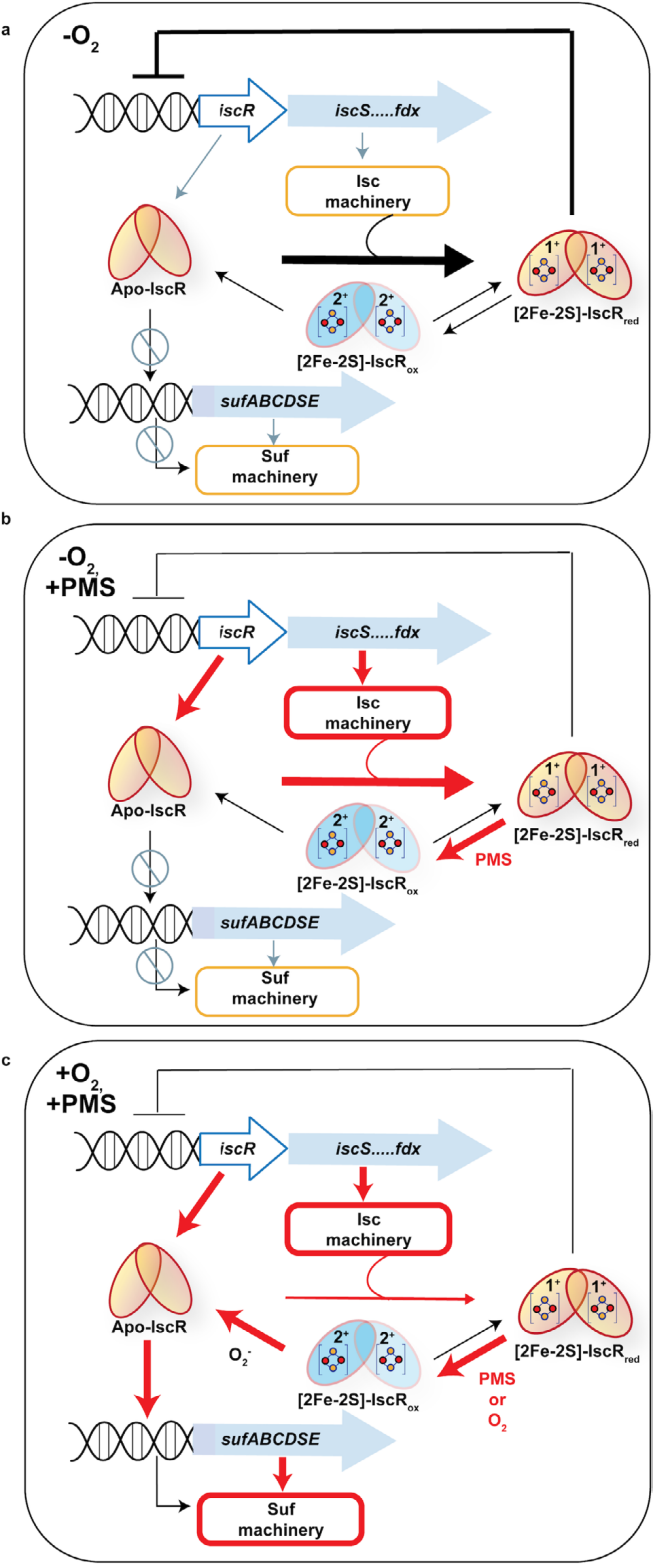
Model for regulation of Fe‐S homeostasis by IscR in response to O_2_ and PMS. (a) [2Fe‐2S]^1+^‐IscR represses expression of the *isc* operon under anaerobic conditions. The *suf* operon is not significantly activated due to low levels of apo‐IscR. (b) The presence of PMS under anaerobic conditions leads to oxidation of the IscR [2Fe‐2S]^1+^cluster to [2Fe‐2S]^2+^ without any significant cluster loss. [2Fe‐2S]^2+^‐IscR is unable to bind to type 1 sites located upstream of the *isc* operon, resulting in derepression of transcription. Similar to anaerobic conditions (a), the *suf* operon is not significantly upregulated because apo‐IscR levels are presumably unchanged by PMS under anaerobic conditions. (c) Under aerobic conditions, either O_2_ or PMS can oxidize the IscR [2Fe‐2S]^1+^ cluster. Additionally, PMS can generate superoxide that causes cluster degradation of some Fe‐S cluster proteins, including IscR, leading to a decrease in the pool of [2Fe‐2S]‐IscR and an increase in the pool of apo‐IscR. Under these conditions, the *isc* operon is further derepressed and the *suf* operon is activated. Presumably, the demand for Fe‐S cluster biogenesis also increases, slowing the synthesis of [2Fe‐2S]^1+^‐IscR from either the cluster degraded apo‐IscR or newly synthesized IscR produced from the derepressed *isc* operon. Unknown reducing systems likely participate to convert [2Fe‐2S]^2+^‐IscR to [2Fe‐2S]^1+^‐IscR to restore repression of the *isc* operon when the oxidative stress is mitigated. Red arrows or boxes indicate proposed increases relative to anaerobic conditions.

Previous studies showed that apo‐IscR, not [2Fe‐2S]^1+^‐IscR, is the more effective transcriptional regulator of promoters containing type 2 sites, despite binding with similar affinities (Nesbit et al. 2009; Chowdhury et al. 2021). The fact that we did not observe PMS‐dependent transcriptional regulation of type 2 promoters under anaerobic growth conditions suggests that oxidized [2Fe‐2S]^2+^‐IscR is also not an effective regulator of transcription. These results also support the notion that apo‐IscR does not accumulate under anaerobic conditions with PMS treatment (Figure 8). However, apo‐IscR does appear to accumulate under aerobic conditions following PMS treatment since expression of type 2 site containing promoters was enhanced by PMS treatment beyond the normal changes in expression brought about by aerobic conditions.

The mechanisms that lead to the PMS‐dependent increase in apo‐IscR under aerobic growth conditions are likely to be multifaceted (Figure 8). Previously, we described a homeostatic model to explain the central role of IscR in transcriptional regulation of both the ISC and SUF pathways by modulating levels of apo‐IscR and [2Fe‐2S]^1+^‐IscR (Giel et al. 2013). We extend this model to include that oxidized [2Fe‐2S]^2+^‐IscR is also ineffective in repressing the *isc* promoter under aerobic conditions, and this leads to the upregulation of the *isc* operon and increased IscR levels. The expected increased demand for Fe‐S cluster biogenesis under aerobic conditions leads to a corresponding decrease in the synthesis of [2Fe‐2S]^1+^‐IscR, a shift in the IscR pool to the apoprotein state, and increased regulation of *suf* and other type 2 promoters. Because PMS can also oxidize [2Fe‐2S]^1+^‐IscR, in addition to O_2_ under aerobic conditions, the additional derepression of the *isc* operon will likely increase apo‐IscR levels by the same mechanism.

However, some apo‐IscR may be formed from degradation of the [2Fe‐2S]^2+^ cluster by superoxide (Figure 8, bottom panel). Our in vitro results show that superoxide, and to a lesser extent H_2_O_2_, accelerated [2Fe‐2S]^2+^ cluster degradation as compared to O_2_ alone. Since superoxide is produced upon PMS treatment of cells (Hassan and Fridovich 1979), our in vitro results suggest that redox‐cycling agents could indirectly contribute to the pool of apo‐IscR via superoxide mediated cluster destruction following oxidation. Indeed, both a strain that produces high endogenous levels of superoxide (*sodAsodB*) and a strain treated with PMS under aerobic conditions (but not anaerobic conditions) increased expression of a type 2 promoter that depends on apo‐IscR. The rate of cluster destruction observed in vitro with superoxide (4.8 × 10^5^ M^−1^ s^−1^) was slower than the rate of superoxide mediated inactivation of dehydratase enzymes (10^6^–10^7^ M^−1^ s^−1^) but faster than the rate of SoxR reactivity (< 1000 M^−1^ s^−1^) (Gu and Imlay 2011) (Flint, Tuminello, and Emptage 1993). Thus, some cluster destruction could contribute to the pool of apo‐IscR depending on the levels of superoxide that accumulate in response to redox‐cycling agents. Finally, we cannot rule out that additional factors may also play a role in the formation of apo‐IscR with redox‐cycling agents. One such factor may be the oxidative stress regulatory sRNA, OxyS, which was recently shown to stabilize *iscR* mRNA (Baussier et al. 2024).

### 3.4 Cluster Oxidation of IscR Has Broad Effects on Bacterial Physiology Under Aerobic Conditions

Bacteria encounter redox‐cycling agents in nature where their effects on redox‐cycling, through depleting NAD(P)H pools and producing superoxide, inhibit bacterial growth (Dietrich and Kiley 2011). For cells to survive such encounters, they must upregulate the appropriate defense mechanisms (Pomposiello and Demple 2002; Blanchard et al. 2007). Our work reinforces previous studies of the important role of IscR in this response (Gerstel et al. 2020; Yeo et al. 2006). Beyond the direct effects of PMS on expression of Fe‐S cluster biogenesis pathways, previous work has shown that upregulation of the SUF pathway in response to PMS conferred resistance to some antibiotics (Gerstel et al. 2020). In addition, the sensitivity of *iscR* null mutants to oxidative stress conditions has been observed with several other bacteria including *Pseudomonas aeruginosa* (Kim et al. 2009; Romsang et al. 2014), *Xanthomonas campestris* (Fuangthong et al. 2015), *Haemophilus influenzae* (Wong et al. 2013), *Vibrio vulnificus* (Lim et al. 2014), and *Dickeya dadantii* (Rincon‐Enriquez et al. 2008). Our data suggest that in * E. coli* this sensitivity likely results from the lack of the IscR dependent upregulation of the *suf* operon. Previous studies have suggested that the SUF pathway functions more robustly than the ISC pathway under oxidative stress conditions (Dai and Outten 2012; Blanc et al. 2014; Jang and Imlay 2010), supporting why apo‐IscR dependent increase in transcription of the *suf* operon is critical under these stress conditions.

In summary, our findings uncovered [2Fe‐2S]^1+^ cluster oxidation as a new step in the regulatory circuitry that controls IscR activity by studying its response to PMS. PMS‐dependent cluster oxidation was found to disable IscR binding to type 1 sites, elevating the expression of the ISC pathway under both aerobic and anaerobic conditions. However, cluster‐oxidized IscR did not enable regulation of type 2 promoters. Rather, our data reinforce the role of a dynamic pool of apo‐IscR in regulating type 2 promoters and propose how PMS treatment impacts this pool under aerobic conditions. While upregulating Fe‐S cluster biogenesis pathways is an appropriate defense mechanism to protect Fe‐S proteins from PMS‐dependent‐mediated destruction, it is noteworthy that PMS and not superoxide is the major input that controls [2Fe‐2S]‐IscR activity. Since redox cycling agents have variable effects on superoxide production and on activating the [2Fe‐2S] SoxR protein, future work should focus on addressing similar questions with IscR.

## 4 Materials and Methods

### 4.1 Strain and Plasmid Construction

Strains and plasmids used in this work are listed in Table 1, and sequences of primers used are available upon request. In‐frame deletions of *sodB*, *rseC*, *rsxC*, *iscS*, *iscR*, and *fpr* were constructed by replacing the coding region with *cat* or *kan* flanked by FLP recognition target (FRT) sites from pKD32 or pKD13, respectively (Datsenko and Wanner 2000). Transduction with P1 *vir* was used to move the *cat* or *kan* allele to appropriate strain backgrounds, and where indicated, *cat* or *kan* was removed by transforming strains with pCP20, encoding FLP recombinase (Datsenko and Wanner 2000). P1 *vir* was also used to move previously described alleles for *sodA* (*sodA25*::Mu*dPR13*; Djaman et al. 2004); P*sufA* (Δ−56 to −51) or (^−50^C to G) relative to the *sufA* transcription start site (Mettert and Kiley 2014); Δ*sufABCDSE* (Outten et al. 2004); and *bla*‐P*tac*‐*iscR(C92A)* (Nesbit et al. 2012). Genotypes were confirmed by colony PCR and DNA sequencing.

**TABLE 1:** Strains and plasmids used in this work^a^.

Strain or plasmid	Relevant genotype or phenotype	Reference or source
**Strains**		
MG1655	λ^−^ F^−^ *rph‐1*	Laboratory stock
PK7747	MG1655 Δ*iscS*	This study
PK4854	MG1655 Δ*iscR*	Schwartz et al. (2001)
PK7571	MG1655 P*iscR*‐*lacZ* (in *lac* region)	Giel et al. (2013)
PK7572	PK4854 P*iscR*‐*lacZ* (in *lac* region)	Giel et al. (2013)
PK16300	PK7571 Δ*rseC* Δ*rsxC*	This study
PK16301	PK7571 Δ*fpr*::*cat*	This study
PK14767	PK7571 *sodA25*::Mu*dPR13* Δ*sodB*	This study
PK6364	MG1655 λP*iscR*‐*lacZ*	Schwartz et al. (2001)
PK6512	PK6364 Δ*iscR*	Giel et al. (2013)
PK6826	PK6364 Δ*fdx*	Giel et al. (2013)
PK12028	MG1655 P*fepB*syn‐*lacZ*	Beauchene et al. (2017)
PK12029	PK12028 Δ*fur*	Beauchene et al. (2017)
PK6879	MG1655 P*sufA*‐*lacZ*	Giel et al. (2006)
PK6880	PK6879 Δ*iscR*	Beauchene et al. (2017)
PK16475	PK6879 *sodA25*::Mu*dPR13* Δ*sodB*	This study
PK10899	PK6879 but with Fur site mutation in P*sufA*‐*lacZ* (^−26^ATA^−24^ changed to ^−26^TAT^−24^)	Mettert and Kiley (2014)
PK11000	PK10899 Δ*iscR*	This study
PK16476	PK10899 *sodA25*::Mu*dPR13* Δ*sodB*	This study
PK8004	MG1655 P*ydiU*‐*lacZ*	(Giel et al. 2006)
PK8005	PK8004 Δ*iscR*	Giel et al. (2006)
PK10554	MG1655 P*hyaA*syn‐*lacZ*, Δ*lacY, bla*‐P*tac*‐*iscR*‐*araC*‐P_*BAD*_‐*iscS*, pACYC184*lacI* ^*q*^‐*tet*	Nesbit et al. (2012)
PK10556	MG1655 P*hyaA*syn‐*lacZ*, Δ*lacY, bla*‐P*tac*‐*iscR(C92A)*‐*araC*‐P_*BAD*_‐*iscS*, pACYC184*lacI* ^*q*^‐*tet*	Nesbit et al. (2012)
PK11291	MG1655 P*sufA*(^−26^ATA^−24^ changed to ^−26^TAT^−24^)‐*lacZ*, Δ*lacY, bla*‐P*tac*‐*iscR*‐*araC*‐P_*BAD*_‐*iscS*, pACYC184*lacI* ^*q*^‐*tet*	Mettert and Kiley (2014)
PK11292	MG1655 P*sufA*(^−26^ATA^−24^ changed to ^−26^TAT^−24^)‐*lacZ*, Δ*lacY, bla*‐P*tac*‐*iscR(C92A)*‐*araC*‐P_*BAD*_‐*iscS*, pACYC184*lacI* ^*q*^‐*tet*	Mettert and Kiley (2014)
PK9523	MG1655 P*iscR*‐*lacZ*, Δ*lacY, bla*‐P*tac*‐*iscR*‐FRT‐*cat*‐FRT‐*araC*‐P_*BAD*_‐*iscS*, pACYC184*lacI* ^*q*^‐*tet*	Giel et al. (2013)
PK9186	MG1655 P*iscR*‐*lacZ*, Δ*lacY, bla*‐P*tac*‐*iscR(C92A)*‐FRT‐*cat*‐FRT‐*araC*‐P_*BAD*_‐*iscS*, pACYC184*lacI* ^*q*^‐*tet*	This study
PK7348	MG1655 Δ*sufABCDSE*::*kan*	This study
PK14715	MG1655 *zdh*‐*3632*::*cat* but Δ bp −56 to −51 for P*sufA*	This study
PK14717	MG1655 *zdh*‐*3632*::*cat* but ^−50^C of P*sufA* changed to G	This study
**Plasmids**		
pKD32	FRT‐*cat*‐FRT	Datsenko and Wanner (2000)
pKD13	FRT‐*kan*‐FRT	Datsenko and Wanner (2000)
pKD46	ori(pSC101) *rep101*(ts)P*araB*‐*gam*‐*bet*‐*exo araC amp*	Datsenko and Wanner (2000)
pCP20	Ap^r^	Datsenko and Wanner (2000)

^a^ Base pair positions are relative to the +1 transcription start site for the promoter described.

### 4.2 β‐Galactosidase Assays

Cultures were grown aerobically, either by shaking (250 rpm) or by sparging with 70% N_2_, 25% O_2_, and 5% CO_2_, or were grown anaerobically by sparging with 95% N_2_ and 5% CO_2_ until cultures reached an OD_600_ of 0.1 to 0.2 at 37°C in either LB medium or morpholinepropanesulfonic acid (MOPS) minimal medium containing 20 mM arabinose, 0.2% casamino acids, and 10 μg/mL tetracycline. In some cases, anaerobic cultures sparged with 95% N_2_ and 5% CO_2_ were shifted to 70% N_2_, 25% O_2_, and 5% CO_2_ using a GB100 PLUS gas mixer (MCQ Instruments). Where indicated, cultures also contained 160 μM IPTG and specified concentrations of PMS. For all assays, a final concentration of 100 μg/mL spectinomycin was added to culture samples to terminate further protein synthesis and cell growth, and cells were kept on ice until assayed for β‐galactosidase activity as previously described (Miller 1972). Assays were repeated at least three independent times, and error bars represent the standard errors of three biological replicates unless otherwise stated.

### 4.3 Purification of IscR

[2Fe‐2S]‐IscR and the apoprotein variants IscR(C92A) and IscR(C92A)His_6_ were isolated anaerobically as previously described (Nesbit et al. 2009; Giel et al. 2006). Protein concentrations were determined using the Bradford Plus protein assay reagent (Pierce) according to the manufacturer's instructions. To determine cluster occupancy, purified [2Fe‐2S]‐IscR was precipitated in an acidic solution (TCA), and the iron content of the cleared solution was determined spectrophotometrically using the TPTZ method (Fischer and Price 1964) and corrected for background iron in the buffer. The percentage of [2Fe‐2S]‐bound IscR was determined assuming 100% occupancy is equivalent to two molecules of iron bound per IscR monomer. The average [2Fe‐2S] cluster occupancy varied between 50% and 70%.

### 4.4 Western Blot Analysis

Cultures were grown at 37°C in LB medium to an OD_600_ of 0.1 and were either left untreated or exposed to 12.5 μM PMS. After an additional 1 h of growth, the final OD_600_ of each culture was measured, aliquots (0.5–1 mL) were centrifuged to pellet the cells, the supernatant was removed, and the pellets were frozen at −80°C. The cell pellets were thawed, resuspended in 50 to 200 μL SDS‐loading buffer, heated for 15 min at 90°C, and 5 to 10 μL of known cell number for each sample were loaded onto a 15% SDS‐polyacrylamide gel for electrophoresis along with aliquots of known amounts of purified IscR(C92A)His_6_, IscS, or IscU. The proteins were then transferred to a nitrocellulose membrane by Western transfer, and IscR, IscS, or IscU levels were detected using rabbit primary antibodies that were raised against the respective proteins and horseradish‐peroxidase‐conjugated goat anti‐rabbit secondary antibodies (Santa Cruz Biotechnology); in the case of α‐IscR and α‐IscS, these antibodies were precleared with lysate from strains PK4854 and PK7747, respectively (Harlow and Lane 1988). After treatment of blots with ECL start Western blotting detection reagent (Amersham), chemiluminescence was quantified using an Azure 600 instrument and ImageJ software. IscR, IscS, and IscU molecules per cell were calculated using the molecular weight for each protein monomer (17.3 kDa for IscR [Schwartz et al. 2001]; 45 kDa for IscS and 17 kDa for IscU [Urbina et al. 2001]) and the number of cells per mL of each culture determined using the conversion factor of OD_600_ of 0.2 = 1.2 × 10^8^ cells per ml. Western blots were performed four times with independently grown cultures. Isolated IscS and IscU and primary antibodies against IscS and IscU were previously described (Urbina et al. 2001).

### 4.5 [2Fe‐2S]‐IscR Cluster Stability Assays

Anaerobic [2Fe‐2S]‐IscR (5 μM) in 10 mM potassium phosphate (pH 7.4), 200 mM KCl was assayed for changes in cluster stability by measuring its absorbance spectra (200 to 700 nm) upon exposure to air or air plus the superoxide generating system of 5 mU of xanthine oxidase and 100 μM hypoxanthine (Sutton et al. 2004) in a 1 mL cuvette every 5 min in a Perkin Elmer Lambda 25 UV/Vis spectrophotometer. Where indicated, 30 U of Cu, Zn superoxide dismutase (Millipore Sigma) was also added. Assays to evaluate cluster stability upon 100 μM H_2_O_2_ exposure were carried out in the same manner as those described for [2Fe‐2S]‐IscR exposure to air. Where indicated, 3600 U of catalase was added. Relative [2Fe‐2S]‐cluster occupancy was calculated as the ratio of A_420nm_ from each spectrum at each time point relative to its value at time *t* = 0 min. For iron binding assays, 1 mM ferene was added to the reaction mix, and the A_593nm_ was monitored over time. Where indicated, 20 μM PMS was added either in the presence or absence of air. Anaerobiosis was achieved by assembling assay components in sealed cuvettes inside a Coy chamber and then, where indicated, immediately equilibrating in air before recording absorbance measurements. All assays were carried out at room temperature and repeated at least three times. The error bars represent standard deviations of three replicate values. Determining the reaction rate of [2Fe‐2S]‐IscR with superoxide is described in Figure S3.

### 4.6 DNA Binding Fluorescence Polarization Assays

[2Fe‐2S]‐IscR was isolated anaerobically as previously described (Giel et al. 2006) with one modification. The Hi‐Prep 16/60 Sephacryl S‐100 HR column fraction was incubated for 15 min with 1 mM sodium dithionite (DTH) before loading onto a BioRex‐70 column equilibrated in 10 mM HEPES (pH 7.4) under anaerobic conditions to remove the DTH. The column was then washed with 5 column volumes of 10 mM HEPES (pH 7.4) 200 mM KCl, and [2Fe‐2S]‐IscR was eluted with 10 mM HEPES (pH 7.4) 1 M KCl. [2Fe‐2S]‐IscR binding to a type 1 (*iscR*B) binding site was measured as previously reported (Rajagopalan et al. 2013). Briefly, 30 bp double‐stranded DNA was generated by annealing (5′‐AAATAGTTGACCATTTTACTCGGGAATGTC‐3′) labeled on the 5′ end with a Texas Red fluorophore (Integrated DNA Technologies) with its complementary strand. Varying concentrations of IscR were incubated in a 500 μL volume of 40 mM Tris‐Cl (pH 7.9), 30 mM KCl, 5% glycerol with or without 2 μM PMS in a Coy anaerobic chamber for 15 min, followed by the addition of DNA and another 15 min incubation before reaction mixes were transferred to glass tubes. The assay tubes were sealed before removing them from the anaerobic chamber for fluorescence polarization measurements. In some cases, [2Fe‐2S]‐IscR was exposed to air for 15 min, and either binding was assayed in air or returned to the anaerobic chamber and incubated at room temperature for 15 min before assaying for DNA binding. All assays were done in triplicate, and error bars represent the standard deviation between replicates.

### 4.7 Electrophoretic Mobility Shift Assays

EMSAs were conducted by incubating 15, 30, and 60 nM of [2Fe‐2S]‐IscR in 40 mM Tris‐Cl (pH 7.9), 150 mM KCl, 10 mM MgCl_2_, and 10% glycerol at room temperature, with or without 240 nM PMS in a Coy anaerobic chamber for 15 min. 4.5 nM of a type 1 site DNA fragment (−300 to +40 bp relative to the *erpA* start codon) was then added, and reaction mixtures in a 10 μL final volume were incubated for 30 min at 37°C. Reaction mixes were loaded onto a 6% native polyacrylamide gel containing 1X running buffer (21 mM MOPS, 5 mM Tris‐Cl [pH 6.6], 5% glycerol) and electrophoresed in 0.5× running buffer for 4 h at 100 V inside the anaerobic chamber. EMSAs conducted with apo‐IscR were carried out in the same manner under anaerobic conditions, except with 3.75, 7.5, and 15 nM of IscR(C92A) and 6 nM of a type 2 site DNA fragment (−200 to +40 bp relative to the *hya* transcription start site). All gels were stained using SYBR Green nucleic acid stain (Invitrogen) and visualized using an Azure 600 imager.

## Author Contributions

**Rajdeep Banerjee:** investigation, writing – review and editing, validation. **Erin L. Mettert:** investigation, writing – original draft, writing – review and editing. **Angela S. Fleischhacker:** funding acquisition, writing – review and editing, investigation. **Patricia J. Kiley:** conceptualization, writing – original draft, writing – review and editing, formal analysis, supervision, project administration, funding acquisition.

## Supporting information


**Data S1:** mmi70021‐sup‐0001‐Supinfo.docx.

## Data Availability

All data are available within the manuscript and Data S1.
