# Supplementary material for: The Role of the [2Fe‐2S] Cluster of Escherichia coli IscR in Responding to Redox‐Cycling Agents
Source: Mol Microbiol. 2025 Sep 11;124(5):433–48. doi: 10.1111/mmi.70021 (PMC12594620; doi:10.1111/mmi.70021)
Supplement: Supplementary file 1 — Data S1: mmi70021‐sup‐0001‐Supinfo.docx. [file MMI-124-433-s001.docx]

SUPPLEMENT TO:

The role of the [2Fe-2S] cluster of *Escherichia coli* IscR in responding to redox-cycling agents

Rajdeep Banerjee^a^, Erin L. Mettert^a^, Angela S. Fleischhacker^a*^, and Patricia J. Kiley^a#^

^a^Department of Biomolecular Chemistry, University of Wisconsin-Madison, Madison, WI, USA

Running Title: IscR and redox-cycling agents

*Present address: Department of Biological Chemistry, University of Michigan Medical School, Ann Arbor, MI, 48109, USA.

^#^Address correspondence to Patricia J. Kiley, [pjkiley@wisc.edu](mailto:pjkiley@wisc.edu)

Rajdeep Banerjee and Erin L. Mettert contributed equally to this work. Author order was determined alphabetically.

Keywords: Fe-S cluster biogenesis, Fe-S cluster homeostasis, Rrf2 family of transcription factors, redox regulation

**
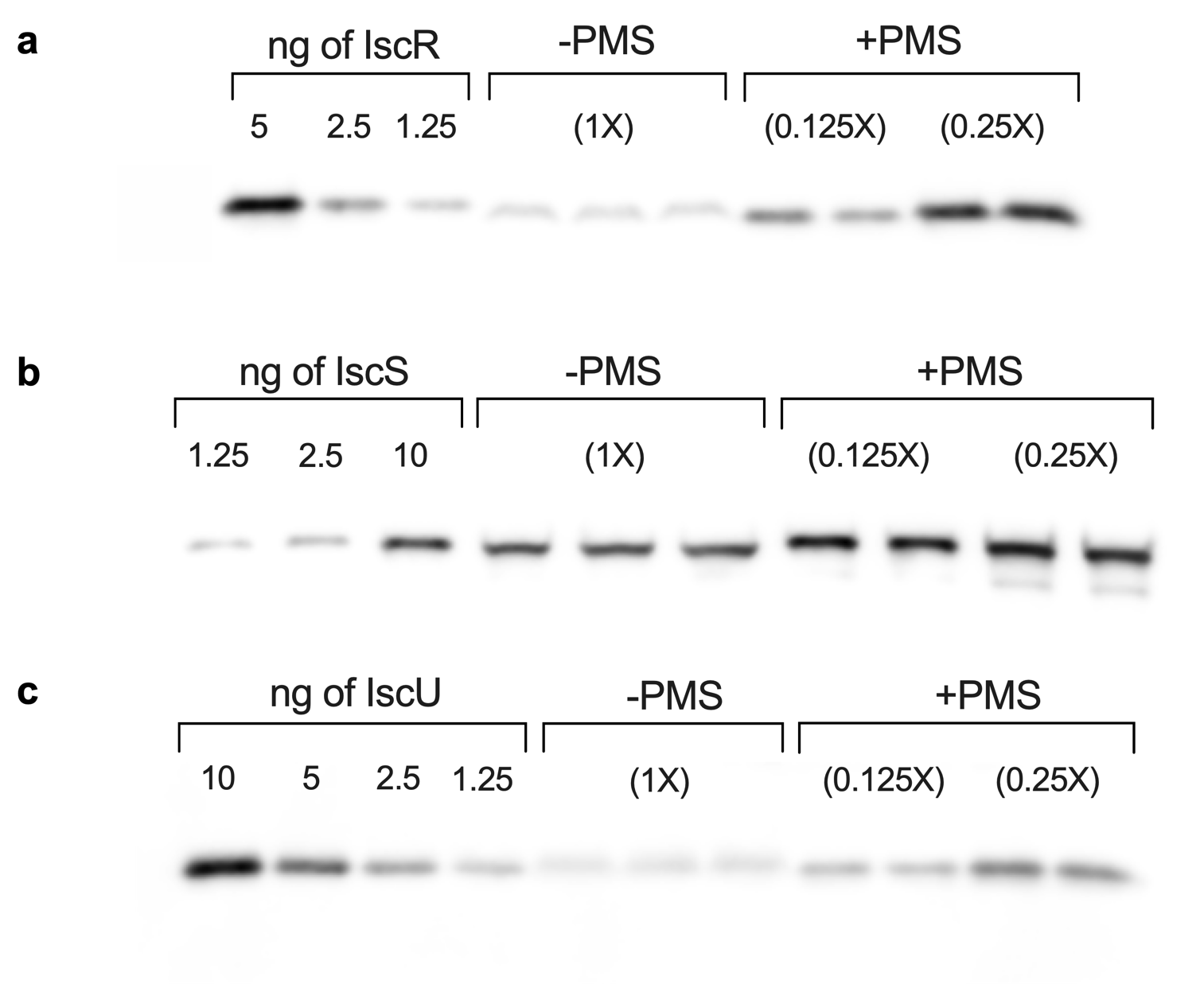
**

**Figure S1.** Western blot analysis was performed to evaluate the effect of PMS on in vivo protein levels of **a**. IscR, **b**. IscS, and **c**. IscU. Cultures of a wild-type strain were grown in LB to an OD_600_ of 0.1 and were either left untreated or exposed to 12.5 μM PMS for 1 h. Triplicate samples of untreated culture and duplicate samples of PMS-exposed cultures, which contained 0.125X and 0.25X the volume of untreated sample, are shown along with known amounts of purified IscR(C92A)His_6_, IscS, or IscU used to generate a standard curve for quantification of protein molecules per cell (Fig. 1).

**
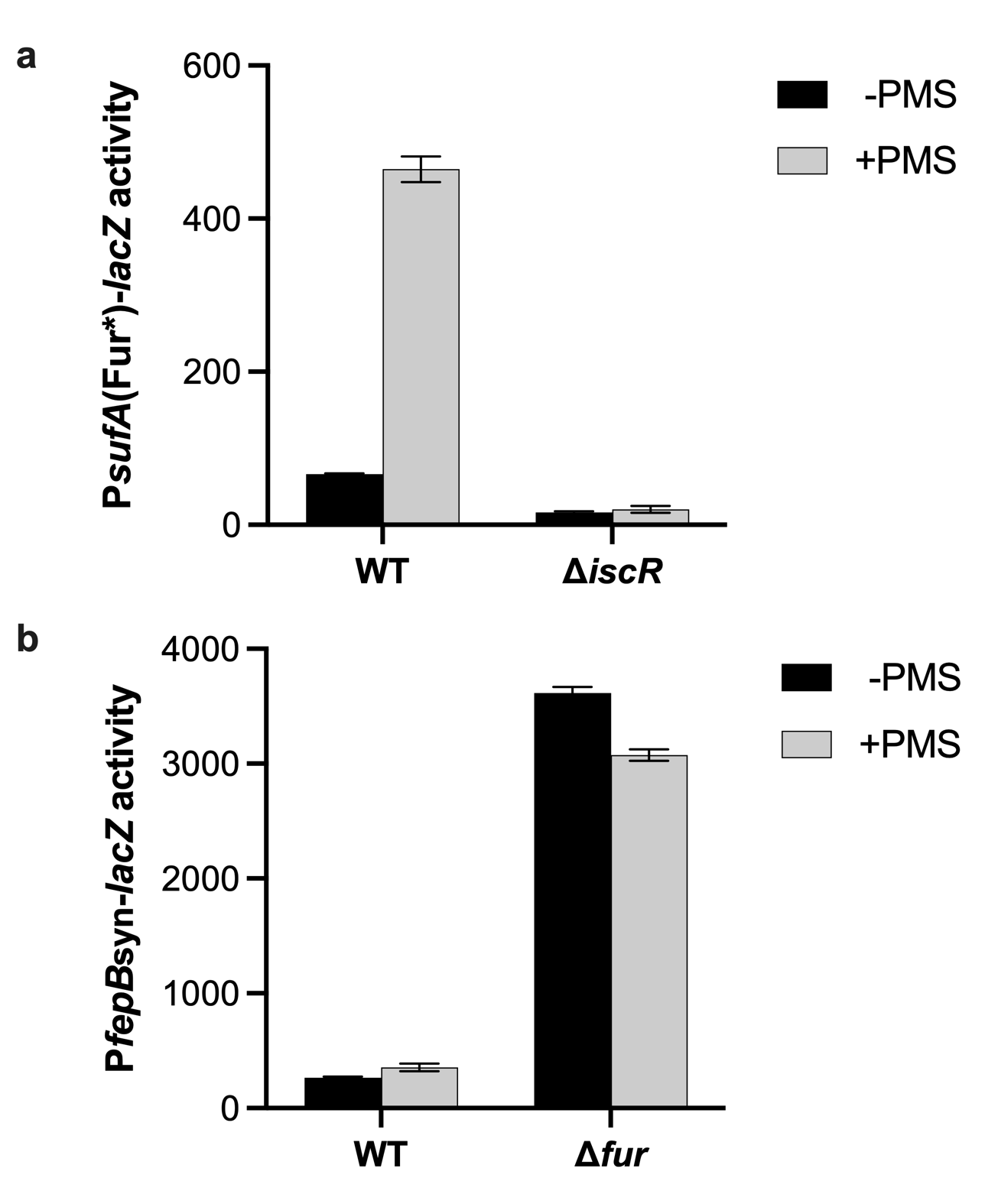
**

**Figure S2.** β-galactosidase activity (Miller units) from **a**. a variant of P*sufA*-*lacZ* in which the Fur binding site was mutated (denoted as Fur*;^-26^ATA^-24^ relative to the transcriptional start site was changed to ^-26^TAT^-24^) and from **b**. a synthetic Fur-repressed promoter reporter (P*fepB*syn-*lacZ*) was measured in wild-type (WT), Δ*iscR*, or Δ*fur* strains. Cultures were grown in LB to an OD_600_ of 0.1 and were either left untreated (black) or exposed to 12.5 μM PMS for 1 h (gray).

**
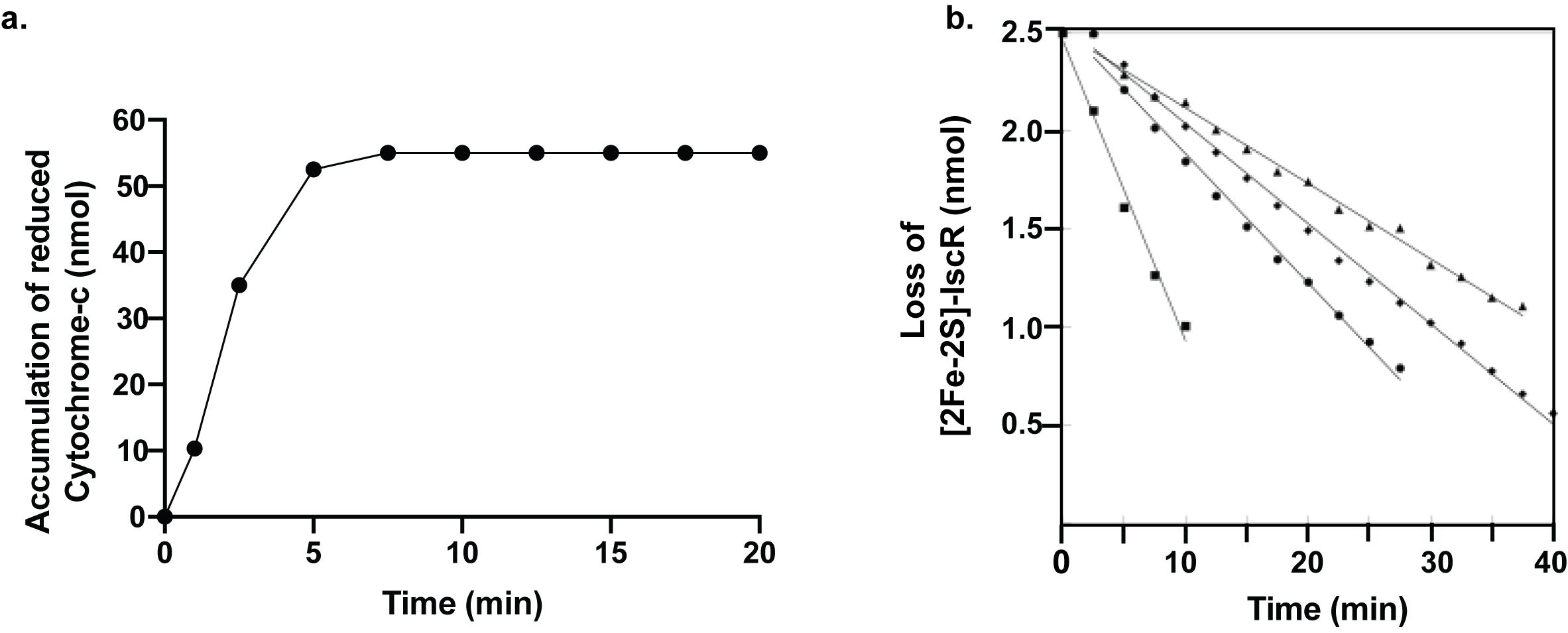
Figure S3:** Determination of the reaction rate of superoxide with IscR. **a.** The rate of superoxide production was measured from the rate of cytochrome c reduction. Cytochrome c (55 μM) was incubated with 200 μM hypoxanthine and 10 mU xanthine oxidase in the presence of 500 U catalase in aerobic buffer [10 mM HEPES (pH 7.4), 200 mM KCl] in 1 ml. Cytochrome c reduction was measured by the absorbance change at 566 nm, using the extinction coefficient 0.021 μM^-1^ cm^-1^ and the rate was calculated from the initial slope and estimated to be 4995 nM min⁻¹.

**b.** Determination of the second-order rate constant for the reaction of IscR with superoxide.
2.5 μM of IscR was incubated in 1 mL under the same conditions as above, in the absence (black box) or presence of CuZn superoxide dismutase (SOD) at concentrations of 0.046 μM (black circle), 0.0615 μM (black prism), and 0.123 μM (black triangle). The rate of [2Fe-2S] cluster degradation of IscR, measured at A_420nm_, was determined from the slopes of the respective curves and calculated to be 153 pmol min⁻¹ (no SOD), 66 pmol min⁻¹ (0.046 μM SOD), 51 pmol min⁻¹ (0.0615 μM SOD), and 38 pmol min⁻¹ (0.123 μM SOD). The second-order rate constant for the reaction between IscR and superoxide was calculated using the method described by Flint et al. (Flint et al., 1993a, Flint et al., 1993b) and estimated to be 4.8 × 10⁵ M⁻¹ s⁻¹.

**
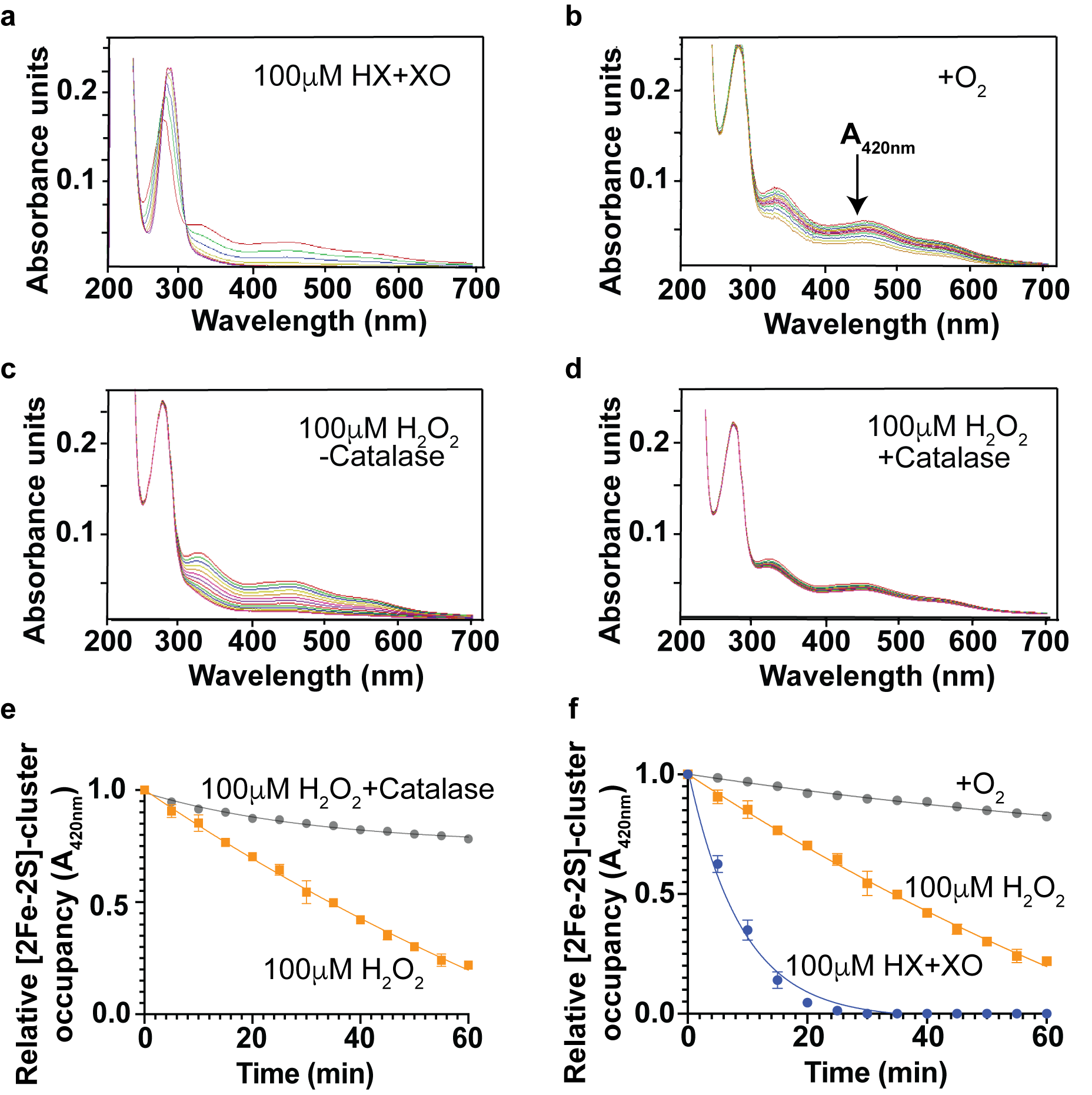
**

**Figure S4.** Exposure of anaerobically isolated [2Fe-2S]-IscR to air, H_2_O_2_ and superoxide. The absorbance spectrum of anaerobic 5 μM [2Fe-2S]-IscR in 10 mM potassium phosphate (pH 7.4) 200 mM KCl was recorded every 5 min after the following treatments: **a.** air, 100 μM hypoxanthine (HX), and 5 mU of xanthine oxidase (XO); **b.** air; **c.** air, 100 μM H_2_O_2_; and **d.** air, 100 μM H_2_O_2_ and 3600 U of catalase. **e.** Relative cluster occupancy of [2Fe-2S]-IscR determined from panels c. and d. was replotted as indicated: air, 100 μM H_2_O_2_ (orange squares); and air, 100 μM H_2_O_2_ and 3600 U of catalase (gray circles). **f.** Data from Fig. 3a, panels b. and c. were replotted to compare the cluster degradation profiles of [2Fe-2S]-IscR under the following conditions: air (gray circles); air, 100 μM hypoxanthine, and 5 mU of xanthine oxidase (blue circles); and air, 100 μM H_2_O_2_ (orange squares).

**
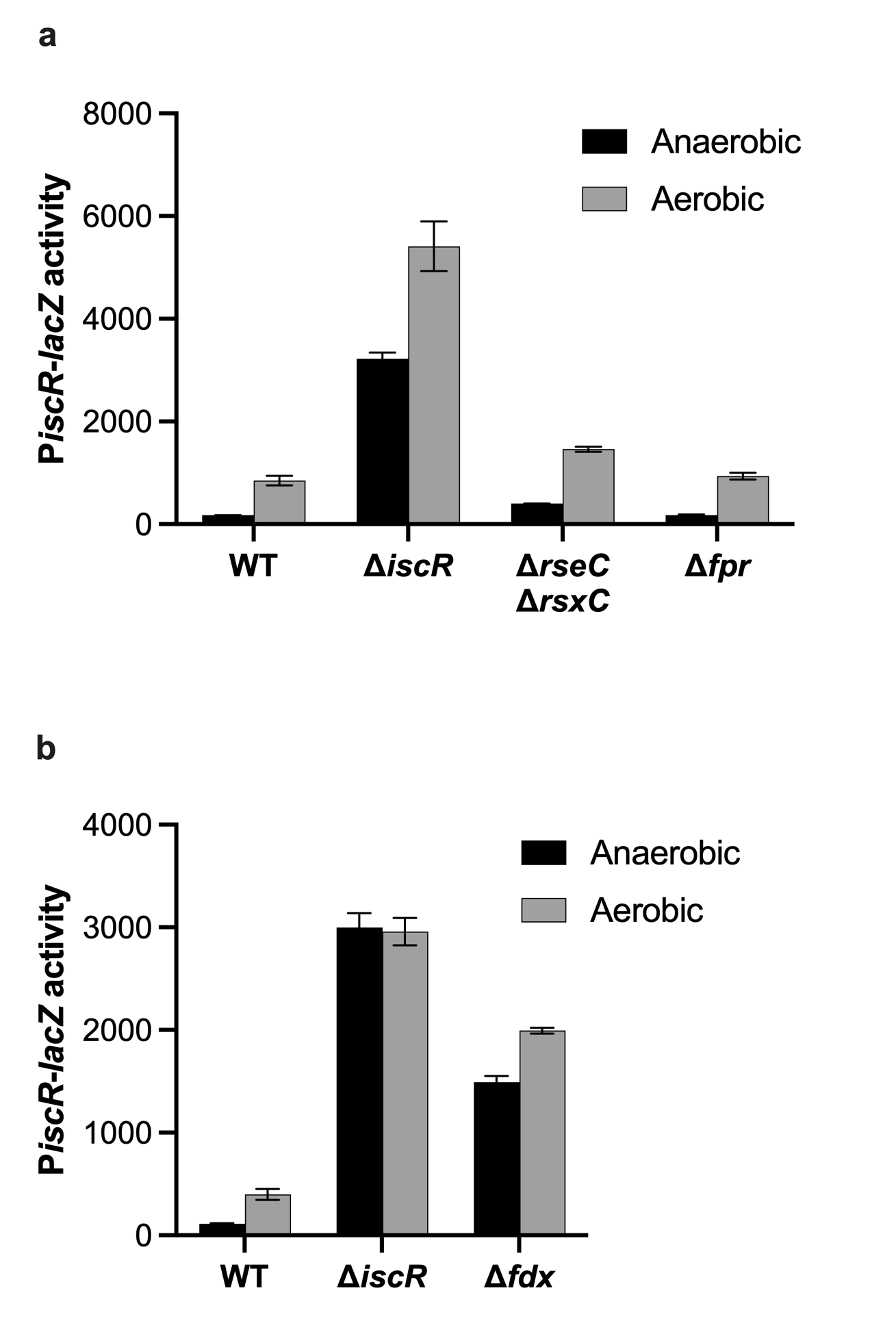
**

**Figure S5**. [2Fe-2S]-IscR activity is defective in a strain lacking ferredoxin encoded by the *isc* operon. β-galactosidase assays were used to measure activity in WT or mutant strains containing P*iscR*-*lacZ* located on the chromosome at either **a**. the *lac* operon or **b**. the λ att site. All cultures were grown in LB under anaerobic (black) or aerobic (gray) conditions.


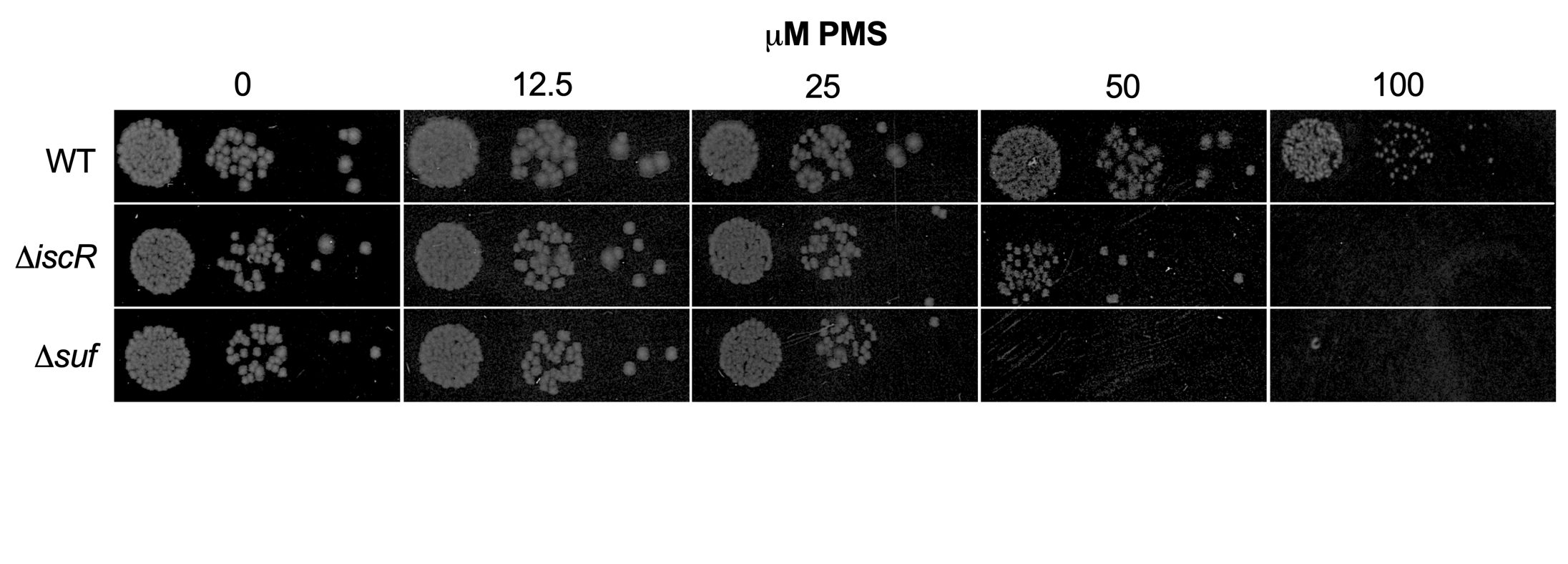
**Figure S6**. IscR and Suf mitigate PMS-induced stress even under anaerobic growth conditions. Serial dilutions of WT or mutant strains grown in LB to an OD_600_ of 0.2 were plated on Tryptone-Yeast Extract (TYE) agar containing various concentrations of PMS. Plates were then incubated ~16 hours at 37°C under anaerobic conditions using a GasPak anaerobic container system (Becton Dickinson).

**Supplementary Table 1**

Cluster Degradation Rate constants (M^-1^s^-1^)

| Proteins | O_2_ | H_2_O_2_ | O_2_^-^ |
| --- | --- | --- | --- |
| IscR | 4.38X10^-2^ | 3.16 | 4.8X10^5^ |
| IscU | 4.8*^a^* | 4.6 *^a^* | - |
| SufB | 0.7 *^a^* | 1.1 *^a^* | - |
| FNR | 180-200*^b^* |  |  |

The rate constant for IscR-[2Fe-2S] cluster degradation with different oxidants was calculated as described in Figure S3 and from the data in Figure S4. IscU*^a^* and SufB*^a^* are from (Blanc et al., 2014) and FNR is from (Crack et al., 2016).

BLANC, B., CLEMANCEY, M., LATOUR, J. M., FONTECAVE, M. & OLLAGNIER DE CHOUDENS, S. 2014. Molecular investigation of iron-sulfur cluster assembly scaffolds under stress. *Biochemistry,* 53**,** 7867-9.

CRACK, J. C., HUTCHINGS, M. I., THOMSON, A. J. & LE BRUN, N. E. 2016. Biochemical properties of *Paracoccus denitrificans* FnrP: reactions with molecular oxygen and nitric oxide. *J Biol Inorg Chem,* 21**,** 71-82.

FLINT, D. H., EMPTAGE, M. H., FINNEGAN, M. G., FU, W. & JOHNSON, M. K. 1993a. The role and properties of the iron-sulfur cluster in *Escherichia coli* dihydroxy-acid dehydratase. *J Biol Chem,* 268**,** 14732-42.

FLINT, D. H., TUMINELLO, J. F. & EMPTAGE, M. H. 1993b. The inactivation of Fe-S cluster containing hydro-lyases by superoxide. *J Biol Chem,* 268**,** 22369-76.
